# hsa_circ_0072389, hsa_circ_0072386, hsa_circ_0008621, hsa_circ_0072387, and hsa_circ_0072391 aggravate glioma via miR-338-5p/IKBIP

**DOI:** 10.18632/aging.203740

**Published:** 2021-12-12

**Authors:** Jian Liang, Xing Li, Jian Xu, Guang-Mou Cai, Jian-Xuan Cao, Bo Zhang

**Affiliations:** 1 The Second Clinical Medical College of Jinan University, Shenzhen People′s Hospital, Shenzhen, China; 2 Department of Hematology, The Second Clinical Medical College of Jinan University, Shenzhen People′s Hospital, Shenzhen, China; 3 School of Medicine, Southern University of Science and Technology, Shenzhen, China

**Keywords:** miR-338-5p, circRNA, IKBIP, ceRNA, glioma

## Abstract

Glioma is a primary intracranial tumor with high morbidity and mortality. We acquired miR-338-5p, which suppresses the development of glioma, from the GEO and CGGA databases. In addition, we predicted that hsa_circ_0072389, hsa_circ_0072386, hsa_circ_0008621, hsa_circ_0072387, and hsa_circ_0072391 could relieve the silencing of IKBIP by miR-338-5p. By analyzing genes related to IKBIP expression, possible pathways affecting glioma were identified. This study provides new ideas for investigating multiple circRNAs in ceRNAs.

## INTRODUCTION

Glioma is the most common primary malignant brain tumor and accounts for approximately half of all intracranial primary tumors [1]. The current effective treatment methods are surgery, radiotherapy, and chemotherapy, the survival of patients posttreatment is still suboptimal [2]. Fortunately, the development of molecular biotechnology provides new ideas for the diagnosis and treatment of glioma.

Circular RNA is a type of noncoding RNA lacking both a 5′ end cap and a 3′ end polytail that induces a circular RNA structure via covalent bonds [3]. Due to their closed circular structure, circRNAs are seldom degraded by exonuclease, which makes them more stable than linear RNAs [4]. An increasing number of studies show that circRNAs participate in the occurrence and development of a variety of cancers [5]. By regulating gene transcription, interacting with proteins, or translating into polypeptides, circRNAs can participate in the regulation of cancer [6, 7]. However, current research mainly regards circRNA as a competing endogenous RNA [8]. As miRNAs can not only silence downstream genes by targeting mRNAs but also bind sites located in circRNAs (miRNA response elements, MREs), circRNAs can prevent the suppression of target genes by competitively binding to miRNAs [9]. For example, Cen J et al. discovered that circular RNA circSDHC sponges adsorb miR-127-3p, which upregulates the expression of CDKN3 and E2F1 and promotes the proliferation and metastasis of renal cell carcinoma [10]. Chen LY et al. revealed that the circular RNA circ-ERBIN promoted colorectal cancer by sponging miR-125a-5p and miR-138-5p, thereby alleviating the silencing of 4EBP-1 [11]. According to multiple studies, we found that a miRNA could be sponged by multiple circRNAs in different cancers. For example, miR-942-5p could be adsorbed by hsa_circ_0015756 in ovarian cancer, circRNA-AKT1 in cervical cancer, and circ-CEP85L in gastric cancer [12–14]. However, studies of the mechanism by which multiple circRNAs regulate miRNAs in the same cancer have not been performed.

In this study, we found that miR-338-5p is differentially expressed between glioma tumor and normal tissues in GSE datasets (GSE139031, GSE25632, GSE103228, GSE65626), indicating that miR-338-5p may have a stable effect on the occurrence or development of glioma. By analyzing the data in several databases, including circBANK, GEO, and circinteractome, we deduced that hsa_circ_0072389, hsa_circ_0072386, hsa_circ_0008621, hsa_circ_0072387, and hsa_circ_0072391 may aggravate glioma by combining with miR-338-5p. In addition, with the circPrimer and circBase databases, we found that these 5 circRNAs all originated from HMGCS1. Moreover, we found that IKBIP may be the target gene of miR-338-5p by analyzing multiple databases, which involved miRwalk, miRDB, TargetScan, GEO, and GEPIA. Therefore, we believe that hsa_circ_0072389, hsa_circ_0072386, hsa_circ_0008621, hsa_circ_0072387, and hsa_circ_0072391 promote the expression of IKBIP by binding with miR-338-5p. Then, we verified the relationship among circRNAs (hsa_circ_0072389, hsa_circ_0072386, hsa_circ_0008621, hsa_circ_0072387, hsa_circ_0072391), miR-338-5p and IKBIP by Western blot. With the Pathcards and GEPIA databases, we inferred that IKBIP may promote the development of glioma NF-κB, JAK/STAT and TGFβ/SMAD signaling pathways. In summary, we believe that hsa_circ_0072389, hsa_circ_0072386, hsa_circ_0008621, hsa_circ_0072387, and hsa_circ_0072391 induce the NF-κB and JAK/STAT pathways to aggravate glioma via miR-338-5p/IKBIP.

## MATERIALS AND METHODS

### Public database collection

High-throughput data on circRNAs, miRNAs and mRNAs in glioma were obtained from the GEO database. The screening standard for circRNA was |logFC| ≥ 1.5 *p* <0.05 and |logFC| ≥ 2 *p* < 0.05 for miRNA and mRNA. The whole screening process was implemented with the limma R package [15].

### Prediction of circRNAs and mRNAs

The prediction of circRNAs binding with miR-338-5p was based on the circBANK database (http://www.circbank.cn/), and mRNAs that targeted miR-338-5p were discovered according to the results from the miRwalk (http://mirwalk.umm.uni-heidelberg.de/), miRDB (http://mirdb.org/), and targetscan databases (http://www.targetscan.org/vert_72/). All these results indicated the presence of conserved 8-mer and 7-mer sites that match the seed region of miR-338-5p [16, 17].

### Prediction of proteins bind to circRNA

The circinteractome database (https://circinteractome.nia.nih.gov/) was employed to reveal proteins that interact with circRNA. In addition, the TargetScan prediction tool enables the prediction and binding sites for RBPs on reported circRNAs that have yet to be mapped [18].

### KEGG and GO Enrichment Analysis

KEGG was used to analyze the pathways involved in mRNAs, and GO was used to analyze molecular function (MF), biological process (BP), and cellular component. All operations were implemented by using the Clusterprofiler R package [19].

### PPI (Protein–protein interaction)

The protein–protein interaction (PPI) network was constructed by the STRING v11 database (https://string-db.org/) [20].

### circRNA secondary structure and MFE structure

The sequences of circRNAs were obtained from circbase (http://circrna.org/) and further entered into the RNAfold database (http://rna.tbi.univie.ac.at/) to acquire the secondary structures and MFE structures of circRNAs.

### Analysis of survival rate

The overall survival time and gene expression of glioma patients were obtained from the GEPIA database (http://gepia.cancer-pku.cn/). Kaplan–Meier plots were used to analyze the relationship between the survival time and gene expression of glioma patients. The hazard ratio [21] and 95% confidence intervals were acquired by the statistical software SPSS 19.0.

### The correlation of gene expression

Pathway-related genes were obtained from the Pathcards database (https://pathcards.genecards.org/), and the correlation between IKBIP and gene expression in glioma was obtained through the GEPIA database.

### Cell culture

The glioma cell Line U251 was cultured in MEM containing 10% fetal bovine serum and 1% glutamine, and the cell culture environment was 5% carbon dioxide in a 37°C incubator.

### RNA extraction, RT–qPCR

RNA was extracted by TRIzol reagent, and RT–qPCR was performed by a 7500 real-time PCR system (Thermo Fisher Scientific). The miR-338-5p inhibitor was obtained from Boshang Biotechnology (Shanghai). Taking GAPDH as a reference, the primer sequences were as follows:

**Table:** 

hsa_circ_0072389:	Left primer: CAGGTGGAGTTGGAGCAGTA
	Right primer: CAGCGGTCTAATGCACTGAG
hsa_circ_0072386:	Left primer: GCTGCTGTCTTCAATGCTGT
	Right primer: TTTGGCCCAATTAGCAGAGC
hsa_circ_0008621:	Left primer: GCTGCCACTCTGTACTCTCT
	Right primer: GGTGTCCTCTCTGAGCTTCA
hsa_circ_0072387:	Left primer: AGACCGCTGCTATTCTGTCT
	Right primer: TCATTCAGCAACATCCGAGC
hsa_circ_0072391:	Left primer: AGACCGCTGCTATTCTGTCT
	Right primer: TCATTCAGCAACATCCGAGC
GAPDH:	Forward: 5′-GGTCGGAGTCAACGGATTTG-3′
	Reverse: 5′-ATGAGCCCCAGCCTTCTCCAT-3′

### Western blot

A BCA Protein Detection Kit (Thermo Scientific™, Shanghai, China) was used to detect the protein concentration. SDS (5×) was added to the total protein, and the mixture was further heated at 100°C for 10 minutes. The protein was isolated by SDS–PAGE and transferred to polyvinylidene fluoride (PVDF) film. Five percent skim milk was sealed at room temperature for 2 hours and further incubated with primary antibody in a shaking bottle at 4°C for 8–12 hours. Then, we washed the film with Tris-buffered saline and Tween 20 (TBST) 3 times, and each wash lasted 10 minutes. After that, the secondary antibody was incubated with film at room temperature for 1 hour and washed with TBST once more 3 times (10 min each time). Proteins were observed by enhanced chemiluminescence.

### Statistical analyses

Student’s *t*-test was used to determine statistically significant differences. When *P* < 0.05, the difference was statistically significant. The statistical analysis software used in this study was SPSS 19.0.

### Data availability statement

The datasets used in the project are available from the corresponding author. The data that support the findings of this study are openly available in GEO at https://www.ncbi.nlm.nih.gov/geo/.

## RESULTS

### miR-338-5p inhibited the progression of glioma

**Figure 1:**
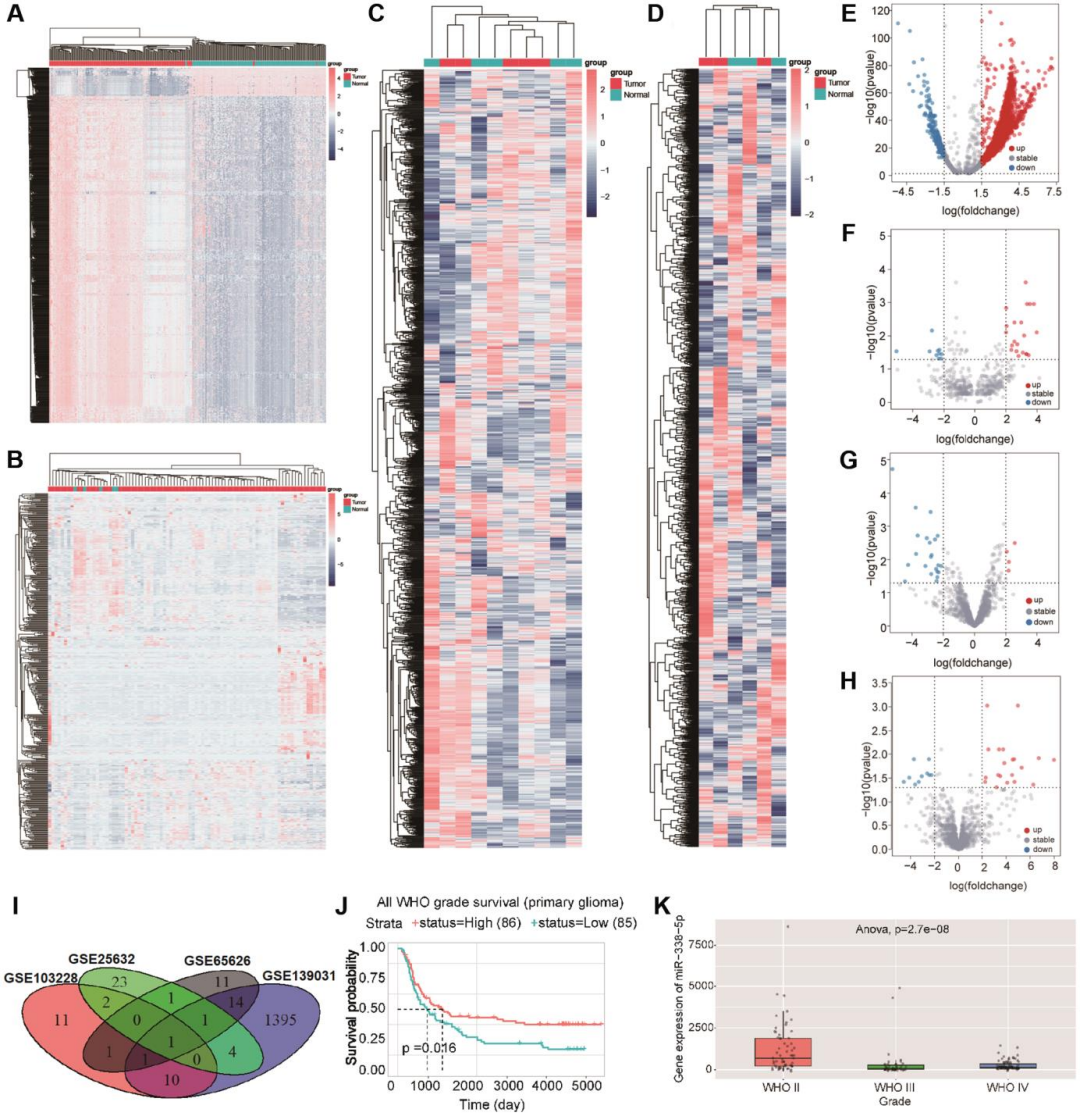
(**A**–**D**) Cluster analysis showed the differential genes in GSE139031, GSE25632, GSE103228, and GSE65626 in turn .red dots indicated upregulated differential genes and blue dots indicated downregulated differential genes. (**E**–**H**) volcano map showed the differential genes in GSE139031, GSE25632, GSE103228, and GSE65626, Red dots represent differential genes with logFC ≥ 2, *p* < 0.05, blue dots represent differential genes with logFC ≤ 2, *p* < 0.05. (**I**)The Venn diagram shows the intersection of differential genes between GSE139031, GSE25632, GSE103228, and GSE65626. (**J**) Survival analysis demonstrated the effect of miR-338-5p on glioma. (**K**) The box diagram showed that the expression level of miR-338-5p in WHO II was significantly higher than that in WHO III and WHO IV.

A number of studies have shown that miRNAs can promote or prevent the occurrence and development of glioma. In this study, we selected GEO datasets (GSE139031, GSE25632, GSE103228, and GSE65626) with large sample sizes for analysis. When comparing gene expression in tumor tissues with gene expression in normal tissues, there were 3556 differential genes in GSE139031, 1145 differential genes in GSE25632, 6658 differential genes in GSE103228, and 3556 differential genes in GSE65626 (Figure 1A–1D). After screening (|logFC| ≥ 2, *p* < 0.05), we found 1426 differential genes in GSE139031 (1312 upregulated genes and 114 downregulated genes), 32 differential genes in GSE25632 (21 upregulated genes and 11 downregulated genes), 26 differential genes in GSE103228 (4 upregulated genes and 22 downregulated genes), and 30 differential genes in GSE65626 (20 upregulated genes and 10 downregulated genes) (Figure 1E–1H). To identify miRNAs stably expressed in glioma, we intersected these differential genes in four datasets and found that miR-338-5p was the only intersection of the four datasets (Figure 1I). By analyzing the CGGA database data, we found that patients with glioma in the miR-338-5p high expression group had a longer survival time, and the expression of miR-338-5p in patients with WHO2 was significantly higher than those with WHO3-4 (Figure 1J–1K); therefore, we speculate that miR-338-5p can inhibit the progression of glioma.

### hsa_circ_0072389, hsa_circ_0072386, hsa_circ_0008621, hsa_circ_0072387, and hsa_circ_0072391 bind to miR-338-5p in glioma

**Figure 2:**
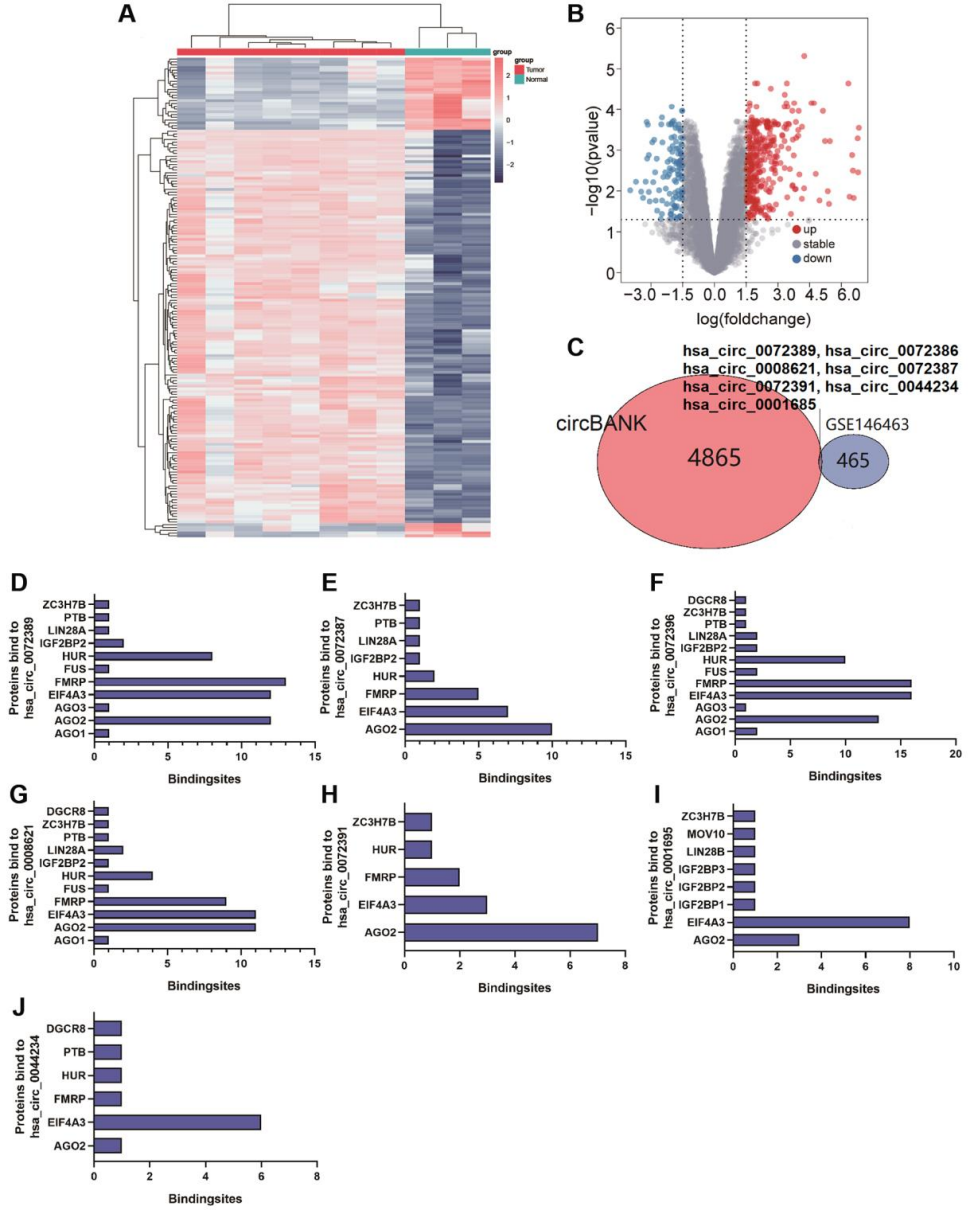
(**A** and **B**) Cluster analysis and volcano map demonstrate differential circRNAs in gliomas of GSE146463. (**C**) Venn Diagram shows circRNAs bound to miR-338-5p. (**D**–**J**) The bar graph shows proteins and the number of binding sites bound to hsa_circ_0072389, hsa_circ_0072386, hsa_circ_0008621, hsa_circ_0072387, hsa_circ_0072391, hsa_circ_0044234, hsa_circ_0001685.

circRNA can target miRNA to prevent the degradation of mRNA induced by miRNA [22]. To explore circRNAs binding to miR-338-5p, 4872 circRNAs were screened out by using circBANK. In addition, we revealed that 472 circRNAs in GSE146463 (|logFC| ≥ 1.5, *p* < 0.05) were differentially expressed between glioma tissues and normal tissues, which included 351 upregulated circRNAs and 121 downregulated circRNAs (Figure 2A and 2B). We found that hsa_circ_0072389, hsa_circ_0072386, hsa_circ_0008621, hsa_circ_0072387, hsa_circ_0072391, hsa_circ_0044234, and hsa_circ_0001685 were not only differentially expressed in GSE146463 but also had separate binding sites for miR-338-5p (Figure 2C). In addition, binding sites for miR-338-5p located in these 7 circRNAs were analyzed in the CircInteractome database (Supplementary Figure 1A–1G). AGO2 is the core component of RISC [21], which connects target sites of miRNA and mRNA [23]. The formation of a ternary complex of circRNA-miRNA-AGO2 can also prove that circRNA interacts with miRNA [24]. We predicted the proteins that may bind to hsa_circ_0072389, hsa_circ_0072386, hsa_circ_0008621, hsa_circ_0072387, hsa_circ_0072391, hsa_circ_0044234, and hsa_circ_0001685 by searching the CircInteractome database and obtained the number of binding sites with AGO2 (Figure 2D–2J). To screen for possible circRNAs binding to miR-338-5p, we selected hsa_circ_0072389, hsa_circ_0072386, hsa_circ_0008621, hsa_circ_0072387, and hsa_circ_0072391, which had great than 5 binding sites with AGO2, for further research.

### Composition, secondary structure and sequence information of circRNAs

Most circRNAs are composed of exons, followed by introns and exons, and few are composed of introns [25]. We obtained the composition and sequence of hsa_circ_0072389, hsa_circ_0072386, hsa_circ_0008621, hsa_circ_0072387, and hsa_circ_0072391 from the circPrimer and circBase databases (Supplementary Figure 1H–1L, Supplementary Figure 2). In addition, the secondary structure and minimum free energy (MFE) structure of circRNAs were acquired from the RNAfold database (Supplementary Figure 3A–3E). Interestingly, we found that hsa_circ_0072389, hsa_circ_0072386, hsa_circ_0008621, hsa_circ_0072387, and hsa_circ_0072391 were all transcribed by the same host gene HMGCS1 on chromosome 5, and they all contained EXON4 and EXON5 (Table 1, Supplementary Figure 1H–1L). In addition, 5 circRNAs were composed of single-stranded structures, stems, hairpin loops, convex loops, inner loops, and multibranched loops, which provide directions for further exploration of the genes regulating transcription, interaction with proteins, or translation into polypeptides.

**Table 1:** CircRNAs binding to miR-338-5p.

**circBASEname**	**Gene symbol**	**Location**	**Sequence**
hsa_circ_0072389	HMGCS1	chr5:43294157-43299077	TACTCTCTTAAAGTCACACAAGATGCTACA CCGGCTCTTTCACCATGCCTGGATCACTTC
hsa_circ_0072386	HMGCS1	chr5:43292575-43299077	CTTTGGATGAAGGAGTAGGACTTGTGCATT CAAACATAGCAACTGAGCTCTTTCACCATG
hsa_circ_0008621	HMGCS1	chr5:43292575-43297268	TTCAAACATAGCAACTGAGGGCTTCGTGGG ACACATATGCAACATGCCTATGATTTTTAC
hsa_circ_0072387	HMGCS1	chr5:43294157-43297268	CAAGATGCTACACCGGGGCTTCGTGGGAC ACATATGCAACATGCCTATGATTTTTACAAG
hsa_circ_0072391	HMGCS1	chr5:43295853-43297268	GAGATAAAAATAGTATCTATAGTGGCCTGG AAGCCTTTGGGGCTTCGTGGGACACATATG
hsa_circ_0044234	CDC27	chr17:45247282-45249430	CATTGTTGGGACATGTATATTGTACACTCA GAAGAAGCCTTGTTTTTACTGGCAACCTGT
hsa_circ_0001685	MPP6	chr7:24663284-24690331	ACCAAGTTATAGAGATACCATTACTCCTCA ACAGCAATGCAGCAAGTCTTGGAAAACCTT

### IKBIP binds to miR-338-5p and aggravates glioma

**Figure 3:**
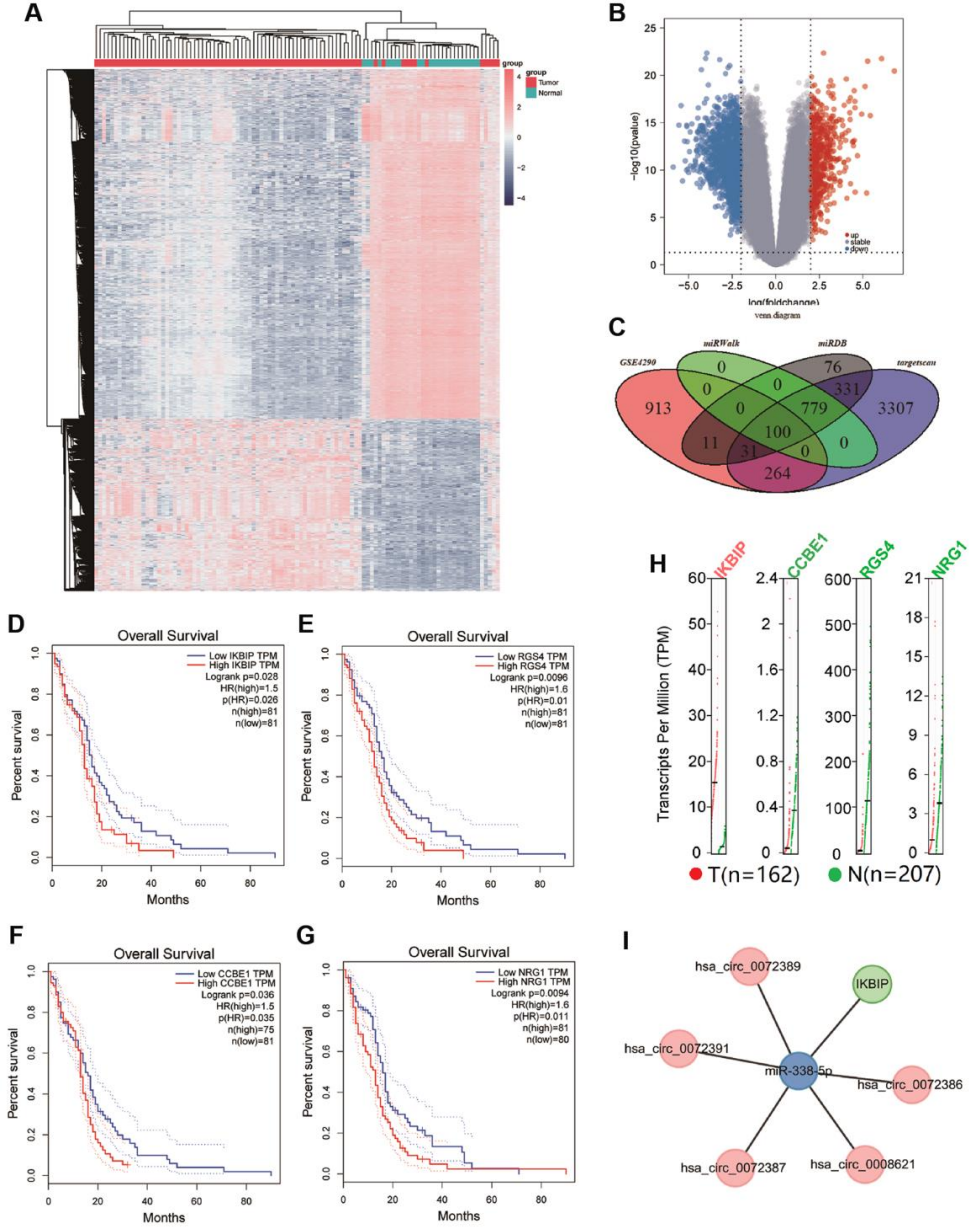
(**A** and **B**) Cluster analysis and volcano map demonstrate differential mRNAs in gliomas of GSE4290. (**C**) The Venn diagram shows the intersection of differential genes between GSE4290, miRwalk database, miRDB database, and targetscan database. (**D**–**G**) Survival curve shown the correlation between the expression of CCBE1, IKBIP, NRG1, and RGS4 with overall survival time (*p* < 0.05). (**H**) Gene Expression Profile of IKBIP, CCBE1, NRG1, and RGS4, red dots indicated the expression of tumor tissue, green dots indicated the expression of normal tissue. (**I**) Network of hsa_circ_0072389, hsa_circ_0072386, hsa_circ_0008621, hsa_circ_0072387, hsa_circ_0072391, miR-338-5p, and IKBIP.

We further predicted mRNAs that might bind miR-338-5p. In GSE4290, we found 1319 differentially expressed genes (|logFC| ≥ 2, *p* < 0.05), which involved 451 upregulated genes and 868 downregulated genes (Figure 3A and 3B). In addition, we obtained 879, 1328, and 4812 mRNAs that may bind to miR-338-5p from the miRwalk, miRDB, and TargetScan databases, respectively. By comparing differential mRNAs in the GSE4290, miRwalk, miRDB, and TargetScan databases, we found that 100 differential mRNAs may not only be correlated with glioma but also bind to miR-338-5p (Figure 3C). Further analysis of these 100 genes by KEGG and GO revealed that these 100 genes tend to regulate signal transduction in pathways, biological processes, cell components, and molecular functions (Supplementary Figure 4A–4D). According to the results described above, we plotted the circRNA-miRNA-mRNA network (Supplementary Figure 5). In addition, using the STRING database, we analyzed the interaction relationships among proteins originating from 100 mRNAs (Supplementary Figure 6). Furthermore, to screen glioma-related mRNAs, we analyzed the effects of these 100 mRNAs on the prognosis of glioma patients in the GEPIA database. We found that patients with low expression of CCBE1, IKBIP, NRG1, and RGS4 had longer survival times (Figure 3D–3G). In addition, we separately acquired the binding sites of CCBE1, IKBIP, NRG1, and RGS4 with miR-338-5p from the TargetScan database (Supplementary Figure 4E–4H). In the GEPIA database, we found that IKBIP was upregulated in glioma relative to normal tissue, while CCBE1, NRG1, and RGS4 were downregulated in glioma (Figure 3H). Since we found that patients with low expression of CCBE1, IKBIP, NRG1, and RGS4 had a better prognosis than patients with high expression, there was a contradiction between the expression of CCBE1, NRG1, and RGS4 and their effect on prognosis. Therefore, we hypothesized that IKBIP was regulated by miR-338-5p in glioma. In summary, we constructed the hsa_circ_0072389, hsa_circ_0072386, hsa_circ_0008621, hsa_circ_0072387, hsa_circ_0072391, miR-338-5p, and IKBIP networks (Figure 3I).

### hsa_circ_0072389, hsa_circ_0072386, hsa_circ_0008621, hsa_circ_0072387, and hsa_circ_0072391 promote the expression of IKBIA, while miR-338-5p has the opposite effect

**Figure 4:**
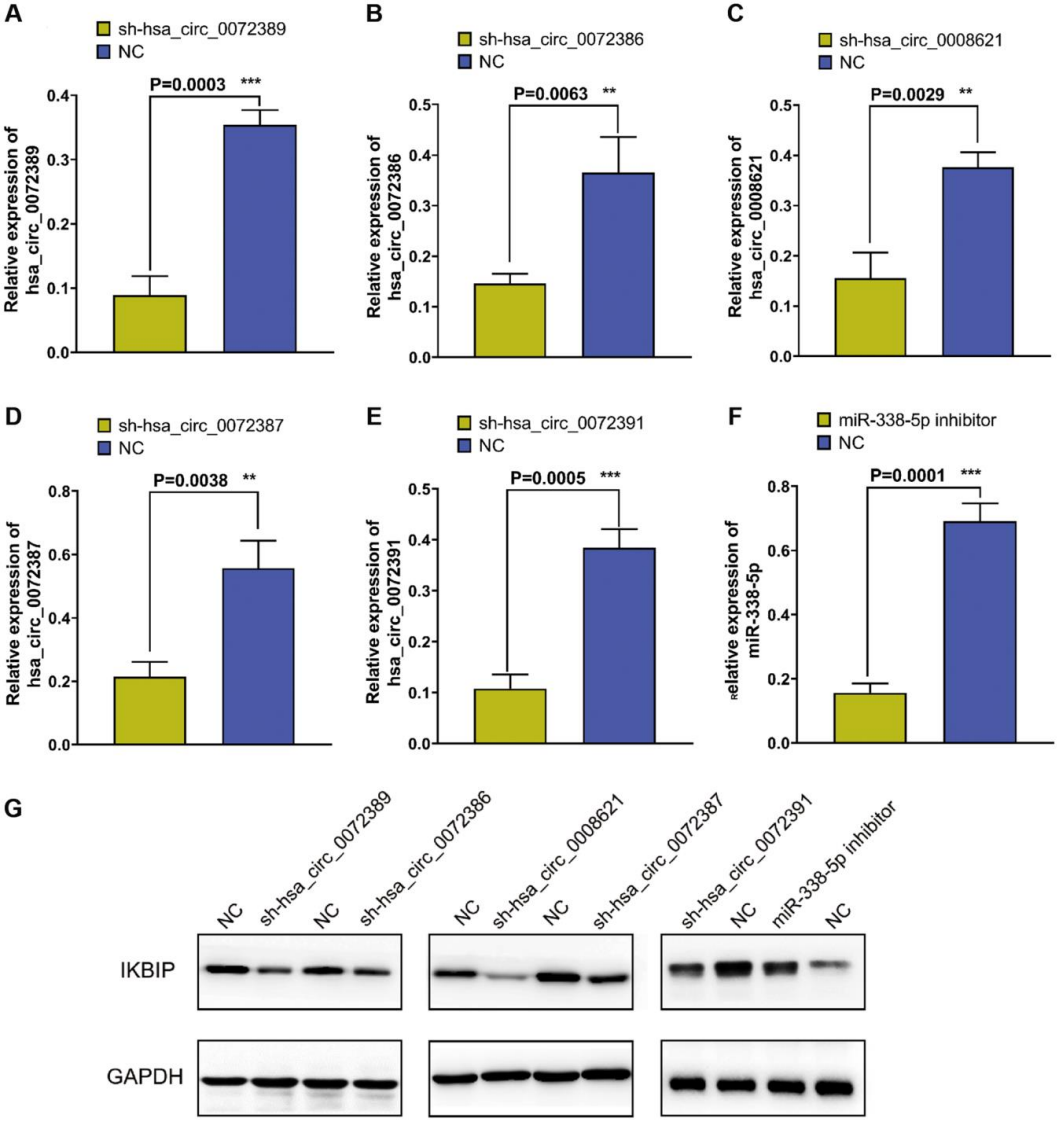
**Figure 4.** (**A**–**E**) RT-qPCR verified the efficiency of knocking down hsa_circ_0072389, hsa_circ_0072386, hsa_circ_0008621, hsa_circ_0072387, hsa_circ_0072391 in glioma U251, respectively. (**F**) RT-qPCR verified the efficiency of miR-338-5p inhibitor on miR-338-5p expression inhibition. (**G**) Western blot shown the effect of hsa_circ_0072389, hsa_circ_0072386, hsa_circ_0008621, hsa_circ_0072387, hsa_circ_0072391, miR-338-5p on IKBIP protein expression.

To verify the relationship among hsa_circ_0072389, hsa_circ_0072386, hsa_circ_0008621, hsa_circ_0072387, and hsa_circ_0072391 with IKBIP, we knocked down hsa_circ_0072389, hsa_circ_0072386, hsa_circ_0008621, hsa_circ_0072387, and hsa_circ_0072391 in U251 glioma cells separately (Figure 4A–4E). Then, Western blotting was used to determine the expression of IKBIP in U251 glioma cells and found that the expression of IKBIP protein was downregulated relative to that in the normal control group (Figure 4G). In addition, we inhibited the expression of miR-338-5p with a miR-338-5p inhibitor (Figure 4F) and revealed that the expression of IKBIP was upregulated relative to that in the normal control group.

### Pathways that may be affected by IKBIP in glioma

**Figure 5:**
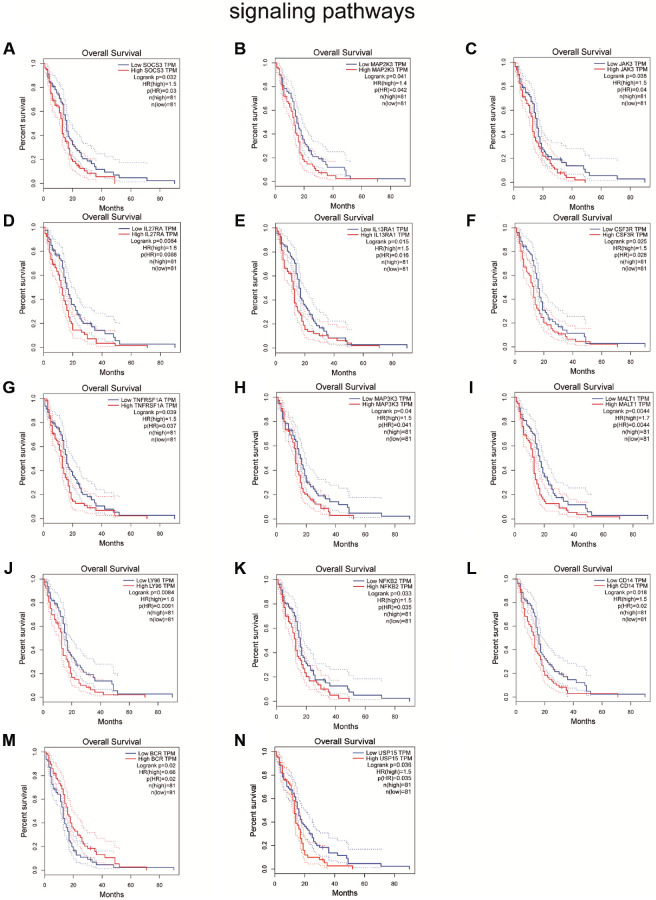
Survival analysis shown the effect of genes related to IKBIP in the (**A**–**F**) JAK/STAT signaling pathway, (**G**–**M**) NF-κB signaling pathway and (**N**) TGFβ/SMAD signaling pathway to glioma patients.

Because genes play different roles in different cancers [26], we explored the pathways that may be affected by IKBIP in glioma by searching the pathcards and GEPIA databases. We searched for genes involved in 8 common pathways (NF-κB signaling pathway, PI3K/Akt signaling pathway, MAPK signaling pathway, JAK/STAT signaling pathway, TGFβ/SMAD signaling pathway, Wnt/β-catenin signaling pathway, Notch signaling pathway, Hedgehog signaling pathway) in the PathCards database and queried the expression of these genes in glioma by analyzing data from the GEPIA database. Then, we found that the expression of the genes correlated with IKBIP (Supplementary Figures 7–14). As not all genes involved in pathways could exert an impact on glioma, we screened significant genes affecting the survival of glioma patients, which included TNFRSF1A (*p* = 5.9 e-9, R = 0.44), MALT1 (*p* = 9.7 e-9, R = 0.37), LY96 (*p* = 1 e-10, R = 0.48), NFKB2 (*p* = 2.5 e-8, R = 0.42), CD14 (*p* = 7 e-8, R = 0.41) in the NF-κB signaling pathway, SOCS3 (*P* = 5 e-8, R = 0.41), MAP2K3 (*p* = 2.4 e-9, R = 0.45), JAK3 (*p* = 1.7 e-5, R = 0.33), IL27RA (*p* = 5.9 e-8, R = 0.41), IL13RA1 (*p* = 3.5 e-13, R = 0.53) in the JAK/STAT signaling pathway, IL27RA (*p* = 5.9 e-8, R = 0.41), and IL13RA1 (*p* = 3.5). These were not only correlated with IKBIP expression (*p* < 0.001, |R| > 0.3) [27] but also affected the prognosis of glioma patients. (Figure 5, Supplementary Figure 15). Therefore, we speculated that IKBIP may induce the development of glioma through the NF-κB signaling pathway, JAK/STAT signaling pathway and TGFβ/SMAD signaling pathway.

### Diagram of circRNA-miRNA-mRNA pathways

**Figure 6:**
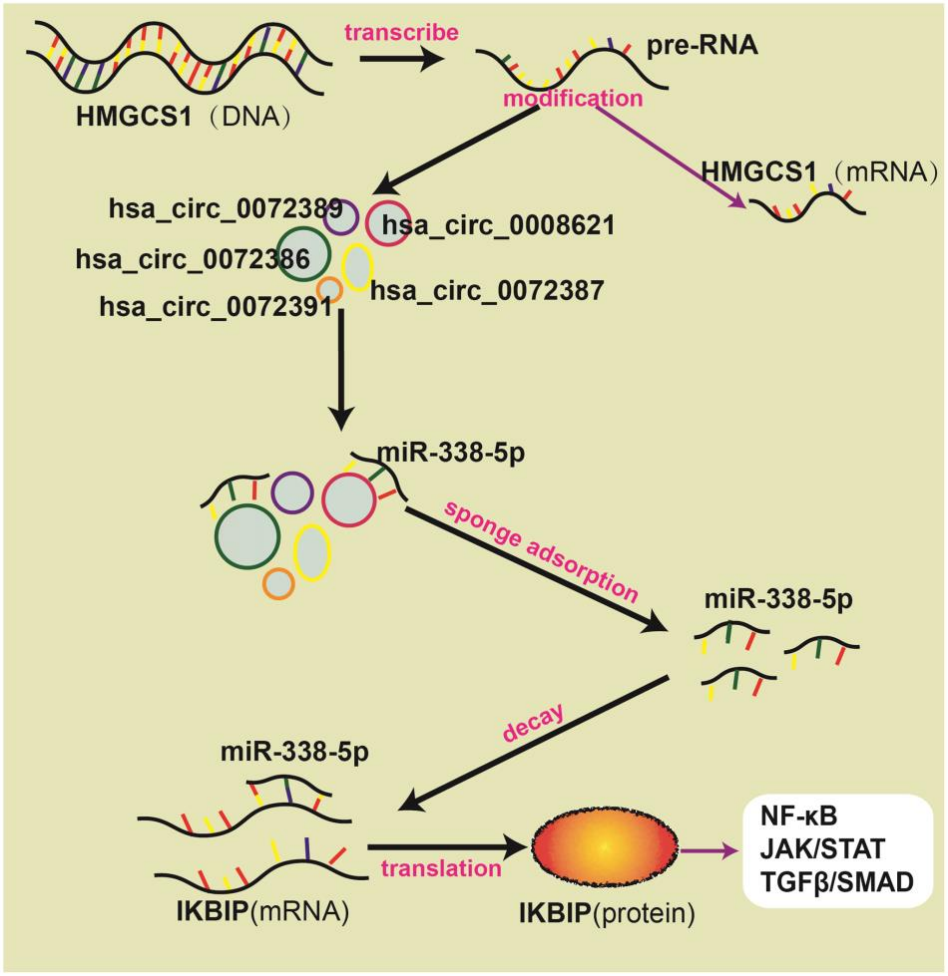
Schematic diagram about ceRNA network.

hsa_circ_0072386, hsa_circ_0008621, hsa_circ_0072387, and hsa_circ_0072391 can bind to miR-338-5p, and miR-338-5p can target IKBIP, which is coexpressed with genes in the NF-κB signaling pathway and JAK/STAT signaling pathway. Therefore, we speculate that after being transcribed from the host gene HMGCS1, pre-RNAs are cleaved into mRNAs and circRNAs through splicing and modification. In these circRNAs, hsa_circ_0072389, hsa_circ_0072386, hsa_circ_0008621, hsa_circ_0072387, and hsa_circ_0072391 are transported between or within cells, which bind to miR-338-5p and hinder the silencing of IKBIP mRNA. Then, the upregulation of IKBIP results in the activation of the NF-κB signaling pathway, JAK/STAT signaling pathway and TGFβ/SMAD signaling pathway, which worsen the deterioration of glioma (Figure 6).

## DISCUSSION

In GSE139031, GSE25632, GSE103228, and GSE65626, we found that miR-338-5p was expressed at low levels in glioma. In the CGGA database, we further verified that glioma patients with high miR-338-5p expression had a better prognosis. Meanwhile, a great number of studies have also shown that miR-338-5p is able to inhibit the proliferation and invasion of glioma cells [28–30]. Therefore, miR-338-5p was selected as a candidate gene for subsequent study. By searching for the presence of conserved 8-mer, 7-mer, and 6-mer sites that match the seed region of miR-338-5p, we predicted circRNAs and mRNA to bind with miR-338-5p [16]. After analyzing the GSE146463, circBANK and circInteractome databases, we found that hsa_circ_0072389, hsa_circ_0072386, hsa_circ_0008621, hsa_circ_0072387, and hsa_circ_0072391 in glioma were most likely to target miR-338-5p. Interestingly, we found that hsa_circ_0072389, hsa_circ_0072386, hsa_circ_0008621, hsa_circ_0072387, and hsa_circ_0072391 were transcribed by the same host gene HMGCS1. Moreover, by using the GEPIA, miRwalk, miRDB, and TargetScan databases and GSE4290, IKBIP was found to facilitate the development of glioma and may be inhibited by miR-338-5p, which induced us to establish a circRNA-miRNA-miRNA network. Furthermore, Western blot analysis verified that downregulation of hsa_circ_0072389, hsa_circ_0072386, hsa_circ_0008621, hsa_circ_0072387, and hsa_circ_0072391 decreased the expression of IKBIP, which could be reversed after inhibiting miR-338-5p. To confirm which pathways might be regulated by IKBIP, we selected the PathCards database to identify all genes in eight common cancer-related pathways (NF-κB signaling pathway, PI3K/Akt signaling pathway, MAPK signaling pathway, JAK/STAT signaling pathway, TGFβ/SMAD signaling pathway, Wnt/β-catenin signaling pathway, Notch signaling pathway, Hedgehog signaling pathway). Genes associated with IKBIP expression were screened by the GEPIA database. However, although these screened genes of 8 common cancer-related pathways may be correlated with IKBIP, they may not affect glioma; therefore, we further performed survival analysis of these genes in glioma by using the GEPIA database. We found that genes in the NF-κB, JAK/STAT and TGFβ/SMAD signaling pathways were not only correlated with IKBIP in gene expression but also affected the survival of glioma patients, which indirectly indicated that these 3 pathways (NF-κB, JAK/STAT and TGFβ/SMAD signaling pathways) may interact with IKBIP to affect the progression of glioma. To acquire a more accurate result, the screening criteria for genes related to IKBIP in expression were stricter (*p* < 0.001, |R| > 0.3) [27]. Then, we found that TNFRSF1A (*p* = 5.9 e-9, R = 0.44), MALT1 (*p* = 9.7 e-9, R = 0.37), LY96 (*p* = 1 e - 10, R = 0.48), NFKB2 (*p* = 2.5 e-8, R = 0.42), CD14 (*p* = 7-8 e, R = 0.41) in the NF-κB signaling pathway, SOCS3 (*p* = 5 e-8, R = 0.41), MAP2K3 (*p* = 2.4 e-9, R = 0.45), JAK3 (*p* = 1.7 e-5, R = 0.33), IL27RA (*p* = 5.9 e-8, R = 0.41), and IL13RA1 (*p* = 3.5 e-13, R = 0.53) in the JAK/STAT signaling pathway, and USP15 (*p* = 3.4 e-11, R = 0.49) in the TGFβ/SMAD signaling pathway were satisfactory. Therefore, we speculated that these 3 signaling pathways may interact with IKBIP to affect the progression of glioma directly, supporting the view that hsa_circ_0072389, hsa_circ_0072386, hsa_circ_0008621, hsa_circ_0072387, and hsa_circ_0072391 upregulated IKBIP by sponging the adsorption of miR-338-5p, which further promoted glioma through the NF-κB signaling pathway, JAK/STAT signaling pathway, and TGFβ/SMAD signaling pathway.

In most studies of ceRNAs in tumors, a single circRNA or mRNA could be screened from circRNA chips or mRNA chips. Then, the targeted miRNA could be predicted easily [31]. However, it was not determined whether a circRNA could only bind to a miRNA invariably [22]. Therefore, this study selected one miRNA, miR-338-5p, as the initial research object and analyzed relationships among circRNAs, miRNAs and mRNAs by exploring the circRNAs to which miR-338-5p may bind. In addition, all genes in 8 common pathways were screened to predict pathways that might be affected by IKBIP. We found that circRNAs (hsa_circ_0072389, hsa_circ_0072386, hsa_circ_0008621, hsa_circ_0072387, and hsa_circ_007239) that bind to miR-338-5p in glioma were all transcribed from the same host gene HMGCS1. In addition, these circRNAs all contained exons 4 and 5. Through the GEPIA database, we found that the change in HMGCS1 mRNA expression in glioma was not statistically significant when compared with the control group. Therefore, we speculated that pre-RNA splicing of hsa_circ_0072389, hsa_circ_0072386, hsa_circ_0008621, hsa_circ_0072387, and hsa_circ_007239 increased with the progression of glioma. As a large-scale research study based on bioinformatics analysis, this work inevitably lacks sufficient experimental verification, so we need to confirm its authenticity in future research. In addition, due to the lack of glioma data in the GEO and TCGA databases, follow-up data of patients are insufficient. Furthermore, although we revealed that hsa_circ_0072389, hsa_circ_0072386, hsa_circ_0008621, hsa_circ_0072387, and hsa_circ_007239 all originate from the same host gene HMGCS1, limited expression data of circRNAs in glioma prevents us from further investigating differences among these five circRNAs. We suspect that the five circRNAs may be differentially expressed not only in diverse grades or prognostic stages of glioma but also in exosomes, cytoplasm or blood, which play significant roles in different times or spaces. In summary, our study provides a novel idea for the study of the complex network among circRNAs-miRNAs-mRNAs, and we also hope to verify the interaction mechanisms among multiple circRNAs, multiple miRNAs, and multiple mRNAs in our future research.

## Supplementary Materials

Supplementary Figures
