# Supplementary material for: hsa_circ_0072389, hsa_circ_0072386, hsa_circ_0008621, hsa_circ_0072387, and hsa_circ_0072391 aggravate glioma via miR-338-5p/IKBIP
Source: Aging (Albany NY). 2021 Dec 12;13(23):25213–40. doi: 10.18632/aging.203740 (PMC8714164; doi:10.18632/aging.203740)
Supplement: Supplementary Figures [file aging-13-203740-s001.pdf]

SUPPLEMENTARY FIGURES

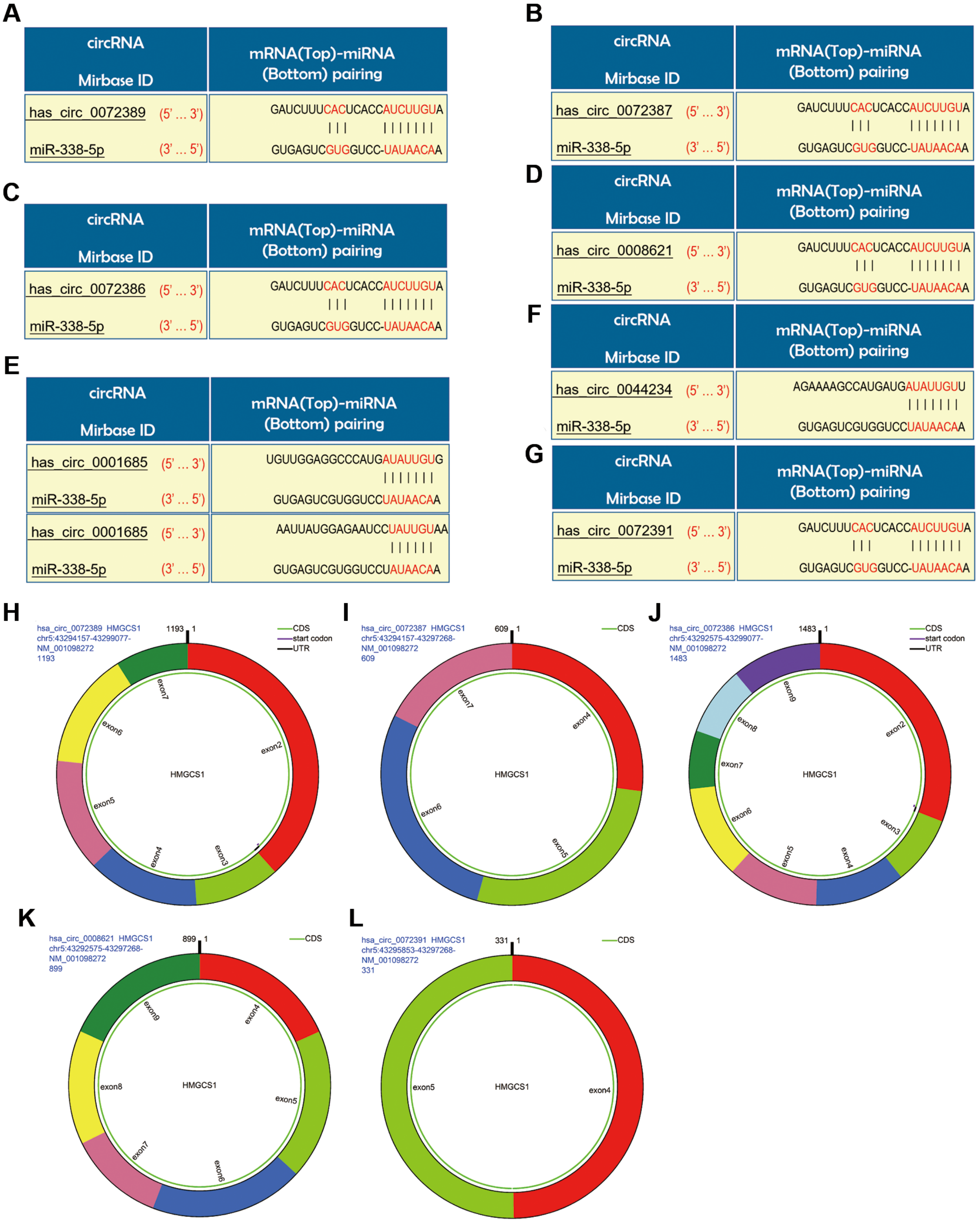

Supplementary Figure 1. (**A**–**G**) Binding site between hsa_circ_0072389, hsa_circ_0072386, hsa_circ_0008621, hsa_circ_0072387, hsa_circ_0072391, hsa_circ_0044234, hsa_circ_0001685 with miR-338-5p. (**H**–**L**) Exon composition, host gene information, chromosome location information of hsa_circ_0072389, hsa_circ_0072386, hsa_circ_0008621, hsa_circ_0072387, hsa_circ_0072391.

hsa_circ_0072389

CTCTTTCACCATGCCTGGATCACTTCCTTTGAATGCAGAAGCTTGCTGGCCAAAAGATGTGGGAATTGTTGCCCTTGAGATCTATTTTCCTTCTCAATATGTTGATCAAGCAGAG
TTGGAAAAATATGATGGTGTAGATGCTGGAAAGTATACCATTGGCTTGGGCCAGGCCAAGATGGGCTTCTGCACAGATAGAGAAGATATTAACTCTCTTTGCATGACTGTGGT
TCAGAATCTTATGGAGAGAAATAACCTTTCCTATGATTGCATTGGGCGGCTGGAAGTTGGAACAGAGACAATCATCGACAAATCAAAGTCTGTGAAGACTAATTTGATGCAGC
TGTTTGAAGAGTCTGGGAATACAGATATAGAAGGAATCGACACAACTAATGCATGCTATGGAGGCACAGCTGCTGTCTTCAATGCTGTTAACTGGATTGAGTCCAGCTCTTGG
GATGGACGGTATGCCCTGGTAGTTGCAGGAGATATTGCTGTATATGCCACAGGAAATGCTAGACCTACAGGTGGAGTTGGAGCAGTAGCTCTGCTAATTGGGCCAAATGCTC
CTTTAATTTTTGAACGAGGGCTTCGTGGGACACATATGCAACATGCCTATGATTTTTACAAGCCTGATATGCTATCTGAATATCCTATAGTAGATGGAAAACTCTCCATACAGTG
CTACCTCAGTGCATTAGACCGCTGCTATTCTGTCTACTGCAAAAAGATCCATGCCCAGTGGCAGAAAGAGGGAAATGATAAAGATTTTACCTTGAATGATTTTGGCTTCATGAT
CTTTCACTCACCATATTGTAAACTGGTTCAGAAATCTCTAGCTCGGATGTTGCTGAATGACTTCCTTAATGACCAGAATAGAGATAAAAATAGTATCTATAGTGGCCTGGAAGC
CTTTGGGGATGTTAAATTAGAAGACACCTACTTTGATAGAGATGTGGAGAAGGCATTTATGAAGGCTAGCTCTGAACTCTTCAGTCAGAAAACAAAGGCATCTTTACTTGTATC
AAATCAAAATGGAAATATGTACACATCTTCAGTATATGGTTCCCTTGCATCTGTTCTAGCACAGTACTCACCTCAGCAATTAGCAGGGAAGAGAATTGGAGTGTTTTCTTATGGT
TCTGGTTTGGCTGCCACTCTGTACTCTCTTAAAGTCACACAAGATGCTACACCGG

hsa_circ_0072387

GGCTTCGTGGGACACATATGCAACATGCCTATGATTTTTACAAGCCTGATATGCTATCTGAATATCCTATAGTAGATGGAAAACTCTCCATACAGTGCTACCTCAGTGCATTAGA
CCGCTGCTATTCTGTCTACTGCAAAAAGATCCATGCCCAGTGGCAGAAAGAGGGAAATGATAAAGATTTTACCTTGAATGATTTTGGCTTCATGATCTTTCACTCACCATATTGT
AAACTGGTTCAGAAATCTCTAGCTCGGATGTTGCTGAATGACTTCCTTAATGACCAGAATAGAGATAAAAATAGTATCTATAGTGGCCTGGAAGCCTTTGGGGATGTTAAATTA
GAAGACACCTACTTTGATAGAGATGTGGAGAAGGCATTTATGAAGGCTAGCTCTGAACTCTTCAGTCAGAAAACAAAGGCATCTTTACTTGTATCAAATCAAAATGGAAATAT
GTACACATCTTCAGTATATGGTTCCCTTGCATCTGTTCTAGCACAGTACTCACCTCAGCAATTAGCAGGGAAGAGAATTGGAGTGTTTTCTTATGGTTCTGGTTTGGCTGCCACT
CTGTACTCTCTTAAAGTCACACAAGATGCTACACCGG

hsa_circ_0072386

CTCTTTCACCATGCCTGGATCACTTCCTTTGAATGCAGAAGCTTGCTGGCCAAAAGATGTGGGAATTGTTGCCCTTGAGATCTATTTTCCTTCTCAATATGTTGATCAAGCAGAG
TTGGAAAAATATGATGGTGTAGATGCTGGAAAGTATACCATTGGCTTGGGCCAGGCCAAGATGGGCTTCTGCACAGATAGAGAAGATATTAACTCTCTTTGCATGACTGTGGT
TCAGAATCTTATGGAGAGAAATAACCTTTCCTATGATTGCATTGGGCGGCTGGAAGTTGGAACAGAGACAATCATCGACAAATCAAAGTCTGTGAAGACTAATTTGATGCAGC
TGTTTGAAGAGTCTGGGAATACAGATATAGAAGGAATCGACACAACTAATGCATGCTATGGAGGCACAGCTGCTGTCTTCAATGCTGTTAACTGGATTGAGTCCAGCTCTTGG
GATGGACGGTATGCCCTGGTAGTTGCAGGAGATATTGCTGTATATGCCACAGGAAATGCTAGACCTACAGGTGGAGTTGGAGCAGTAGCTCTGCTAATTGGGCCAAATGCTC
CTTTAATTTTTGAACGAGGGCTTCGTGGGACACATATGCAACATGCCTATGATTTTTACAAGCCTGATATGCTATCTGAATATCCTATAGTAGATGGAAAACTCTCCATACAGTG
CTACCTCAGTGCATTAGACCGCTGCTATTCTGTCTACTGCAAAAAGATCCATGCCCAGTGGCAGAAAGAGGGAAATGATAAAGATTTTACCTTGAATGATTTTGGCTTCATGAT
CTTTCACTCACCATATTGTAAACTGGTTCAGAAATCTCTAGCTCGGATGTTGCTGAATGACTTCCTTAATGACCAGAATAGAGATAAAAATAGTATCTATAGTGGCCTGGAAGC
CTTTGGGGATGTTAAATTAGAAGACACCTACTTTGATAGAGATGTGGAGAAGGCATTTATGAAGGCTAGCTCTGAACTCTTCAGTCAGAAAACAAAGGCATCTTTACTTGTATC
AAATCAAAATGGAAATATGTACACATCTTCAGTATATGGTTCCCTTGCATCTGTTCTAGCACAGTACTCACCTCAGCAATTAGCAGGGAAGAGAATTGGAGTGTTTTCTTATGGT
TCTGGTTTGGCTGCCACTCTGTACTCTCTTAAAGTCACACAAGATGCTACACCGGGGTCTGCTCTTGATAAAATAACAGCAAGTTTATGTGATCTTAAATCAAGGCTTGATTCAA
GAACTGGTGTGGCACCAGATGTCTTCGCTGAAAACATGAAGCTCAGAGAGGACACCCATCATTTGGTCAACTATATTCCCCAGGGTTCAATAGATTCACTCTTTGAAGGAACG
TGGTACTTAGTTAGGGTGGATGAAAAGCACAGAAGAACTTACGCTCGGCGTCCCACTCCAAATGATGACACTTTGGATGAAGGAGTAGGACTTGTGCATTCAAACATAGCAA
CTGAG

hsa_circ_0008621

ACCTCAGTGCATTAGACCGCTGCTATTCTGTCTACTGCAAAAAGATCCATGCCCAGTGGCAGAAAGAGGGAAATGATAAAGATTTTACCTTGAATGATTTTGGCTTCATGATCT
TTCACTCACCATATTGTAAACTGGTTCAGAAATCTCTAGCTCGGATGTTGCTGAATGACTTCCTTAATGACCAGAATAGAGATAAAAATAGTATCTATAGTGGCCTGGAAGCCTT
TGGGGATGTTAAATTAGAAGACACCTACTTTGATAGAGATGTGGAGAAGGCATTTATGAAGGCTAGCTCTGAACTCTTCAGTCAGAAAACAAAGGCATCTTTACTTGTATCAA
ATCAAAATGGAAATATGTACACATCTTCAGTATATGGTTCCCTTGCATCTGTTCTAGCACAGTACTCACCTCAGCAATTAGCAGGGAAGAGAATTGGAGTGTTTTCTTATGGTTC
TGGTTTGGCTGCCACTCTGTACTCTCTTAAAGTCACACAAGATGCTACACCGGGGTCTGCTCTTGATAAAATAACAGCAAGTTTATGTGATCTTAAATCAAGGCTTGATTCAAGA
ACTGGTGTGGCACCAGATGTCTTCGCTGAAAACATGAAGCTCAGAGAGGACACCCATCATTTGGTCAACTATATTCCCCAGGGTTCAATAGATTCACTCTTTGAAGGAACGTG
GTACTTAGTTAGGGTGGATGAAAAGCACAGAAGAACTTACGCTCGGCGTCCCACTCCAAATGATGACACTTTGGATGAAGGAGTAGGACTTGTGCATTCAAACATAGCAACT
GAG

hsa_circ_0072391

GGCTTCGTGGGACACATATGCAACATGCCTATGATTTTTACAAGCCTGATATGCTATCTGAATATCCTATAGTAGATGGAAAACTCTCCATACAGTGCTACCTCAGTGCATTAGA
CCGCTGCTATTCTGTCTACTGCAAAAAGATCCATGCCCAGTGGCAGAAAGAGGGAAATGATAAAGATTTTACCTTGAATGATTTTGGCTTCATGATCTTTCACTCACCATATTGT
AAACTGGTTCAGAAATCTCTAGCTCGGATGTTGCTGAATGACTTCCTTAATGACCAGAATAGAGATAAAAATAGTATCTATAGTGGCCTGGAAGCCTTTGG

Supplementary Figure 2. The sequence of hsa_circ_0072389, hsa_circ_0072387, hsa_circ_0072386, hsa_circ_0008621, hsa_circ_0072391.

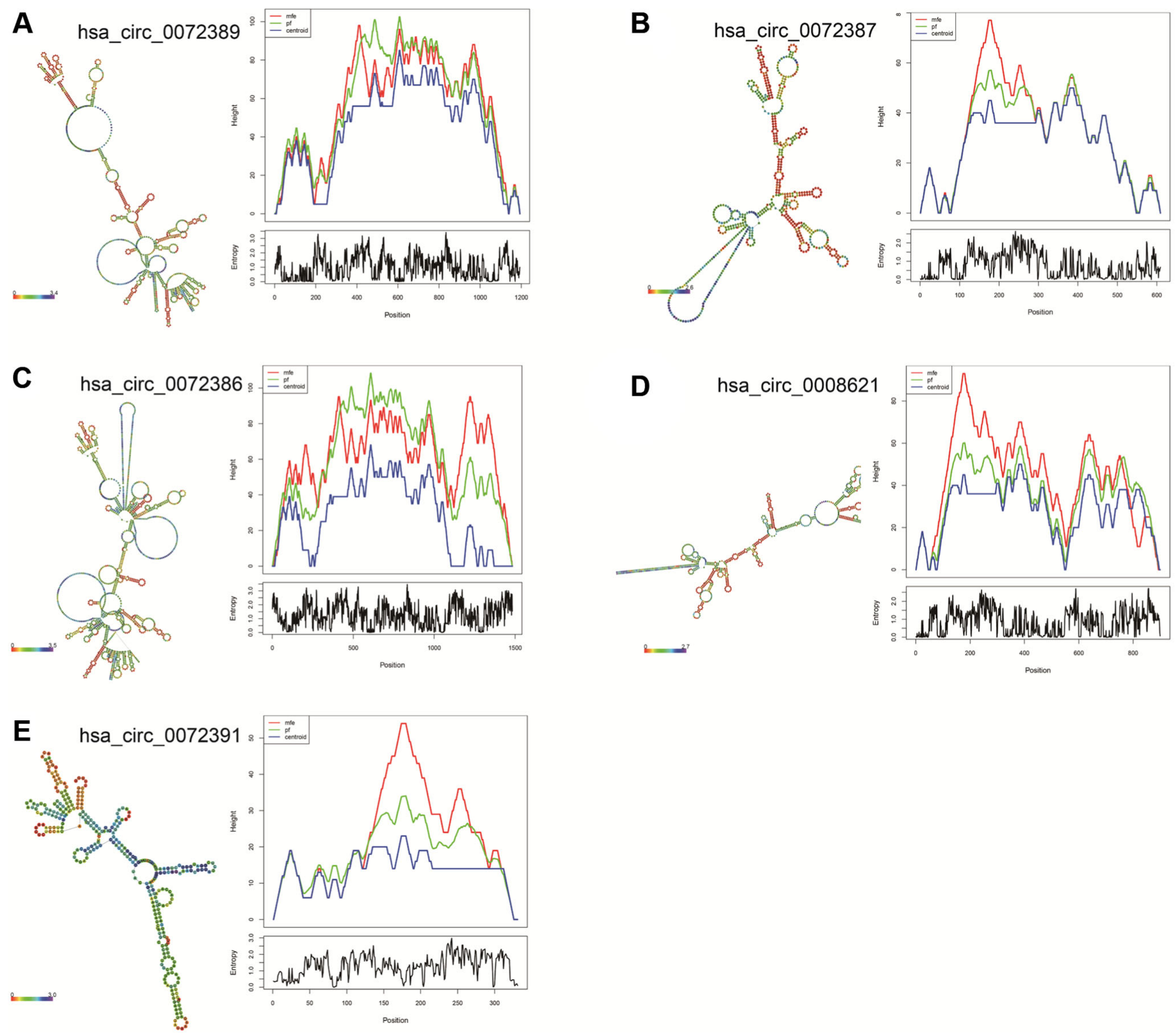

Supplementary Figure 3. The secondary structure and minimum free energy (MFE) structure of (**A**) hsa_circ_0072389, (**B**) hsa_circ_0072387, (**C**) hsa_circ_0072386, (**D**) hsa_circ_0008621, (**E**) hsa_circ_0072391.

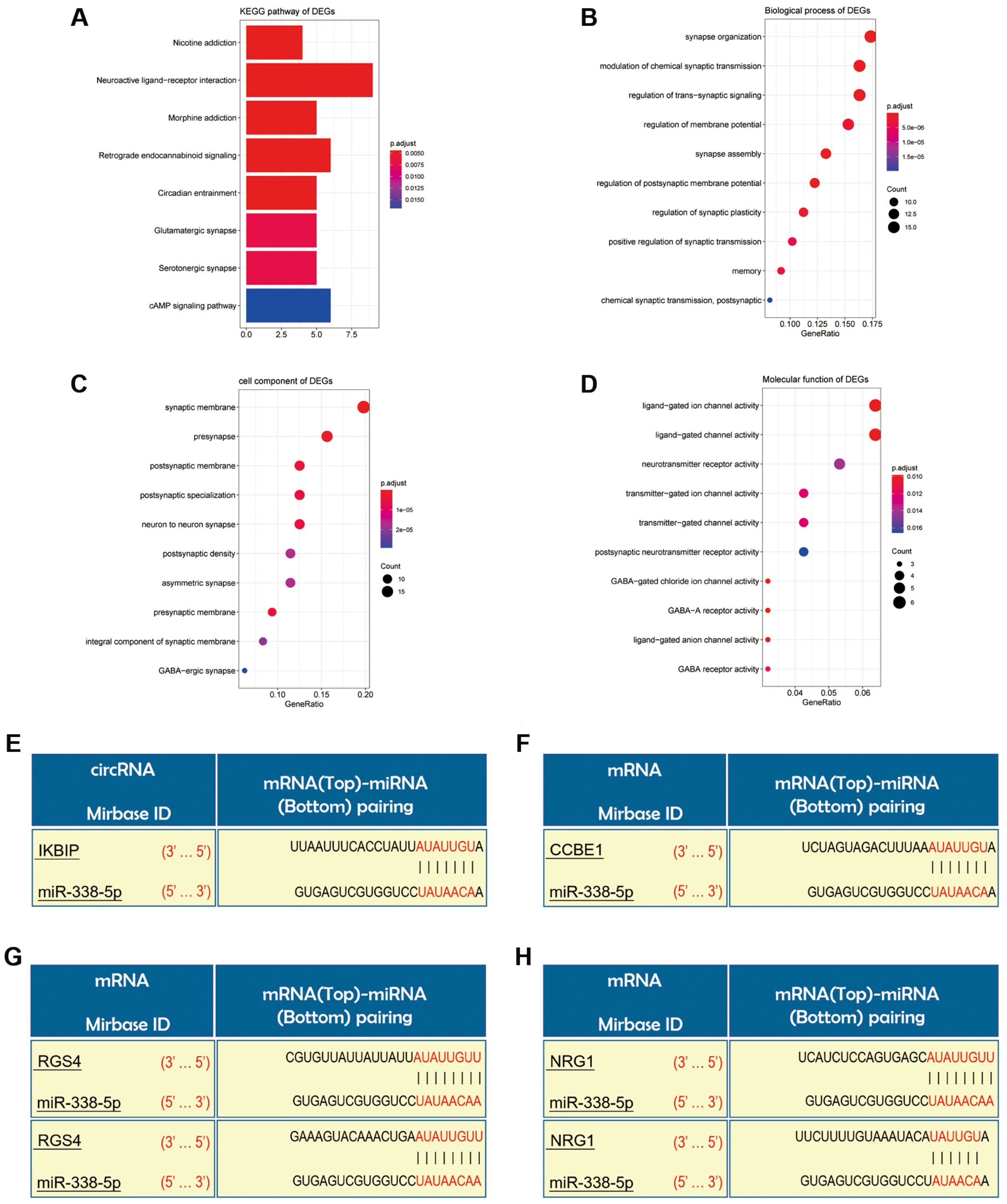

Supplementary Figure 4. (**A**) Pathways involved in the 100 mRNAs analysis by KEGG. (**B**–**D**) Biological process, cell component, and molecular function of the 100 mRNAs by GO analysis. (**E**–**H**) Binding site between CCBE1, IKBIP, NRG1, and RGS4 with miR-338-5p.

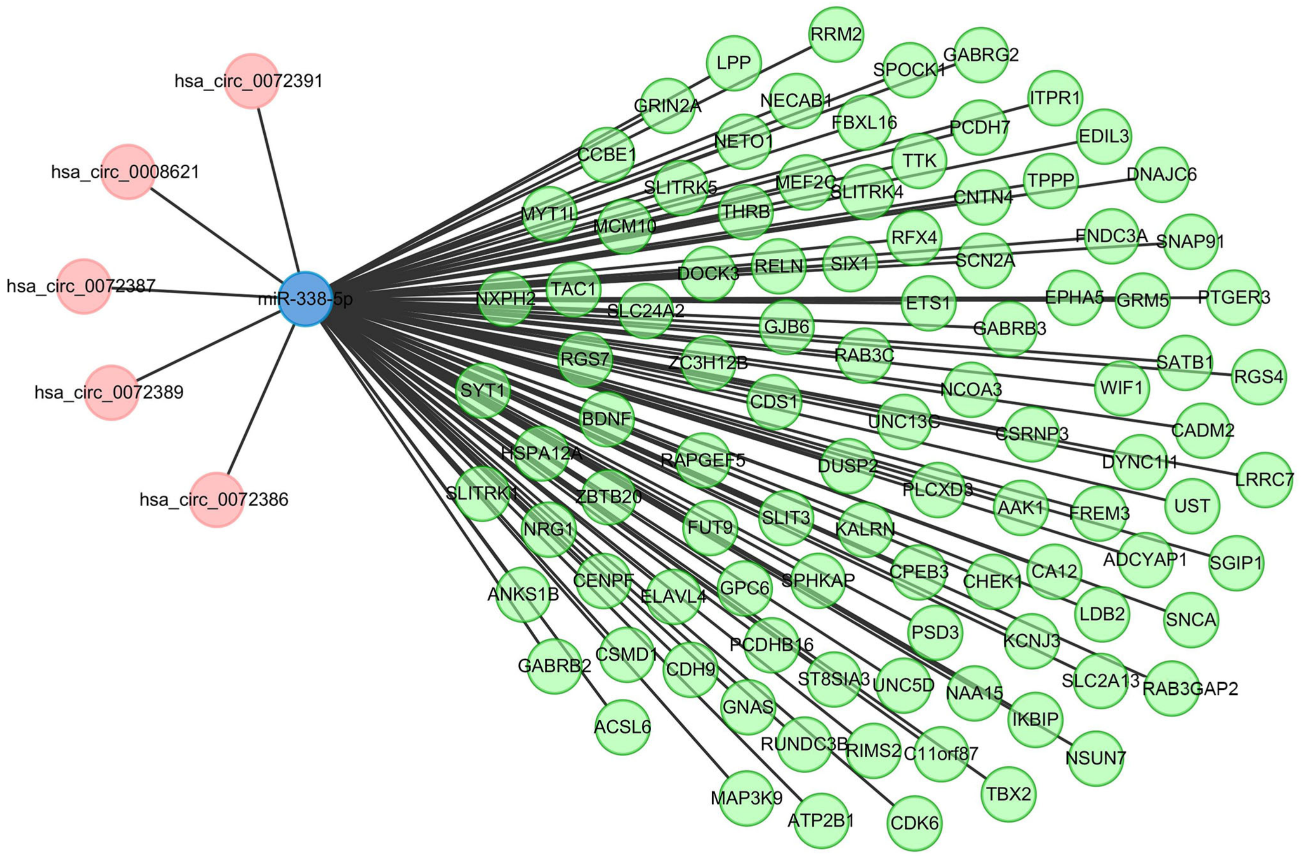

Supplementary Figure 5. circRNA-miRNA-mRNA network about hsa_circ_0072389, hsa_circ_0072386, hsa_circ_0008621, hsa_circ_0072387, hsa_circ_0072391, miR-338-5p, and the 100 mRNAs that might bind to miR-338-5p.

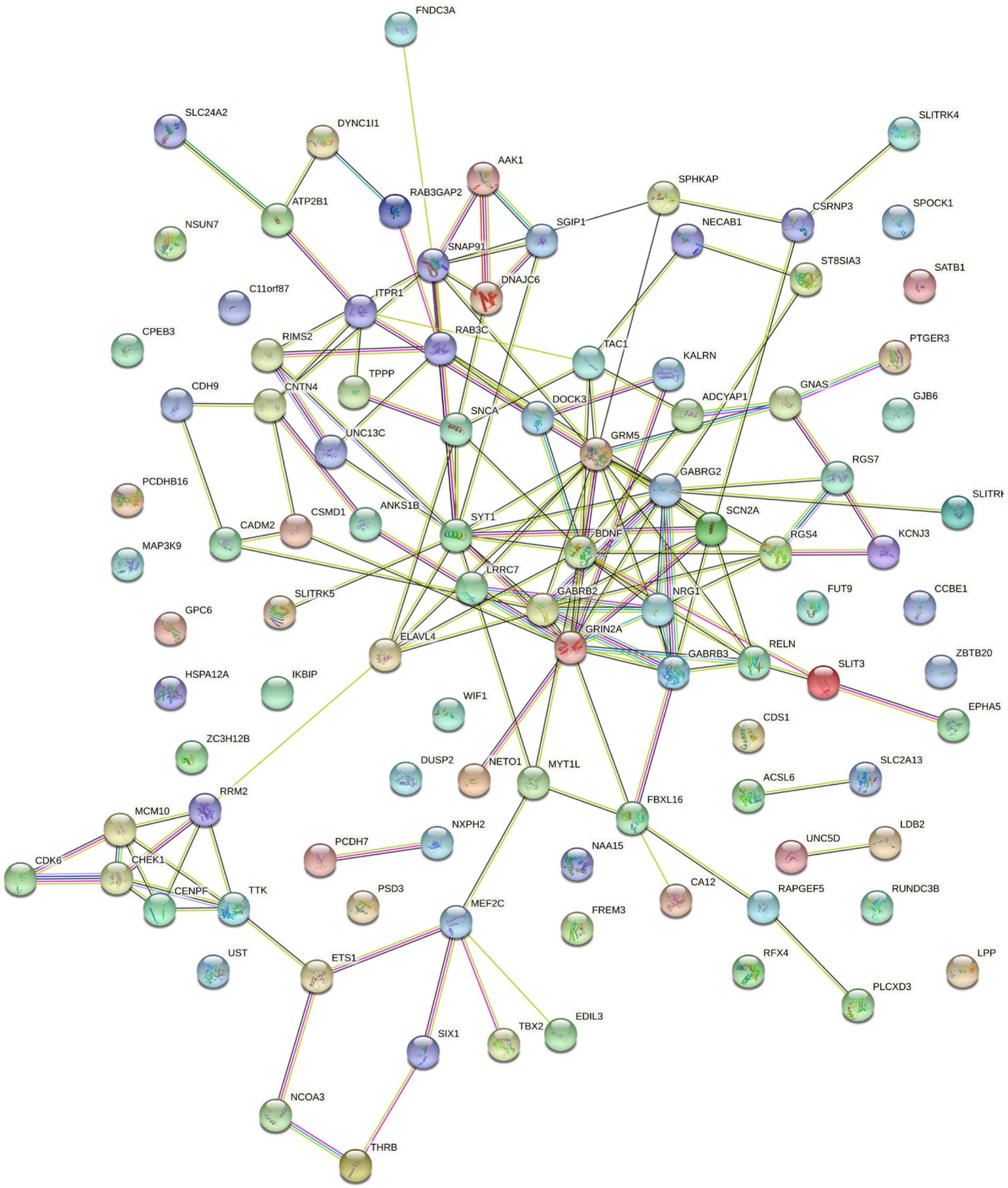

Supplementary Figure 6. PPI network about 100 mRNAs that might bind to miR-338-5p.

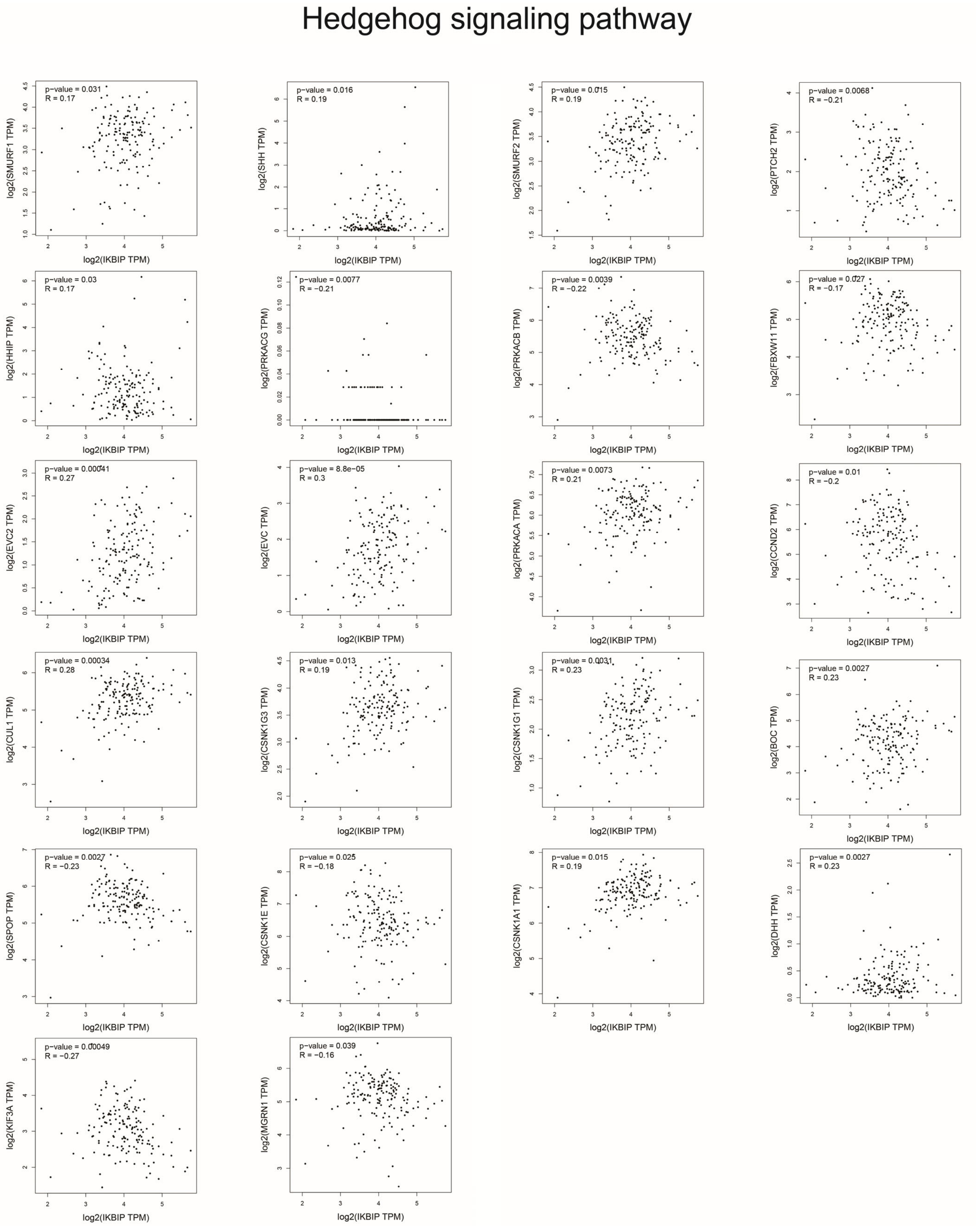

Supplementary Figure 7. Scatter plot shown genes in Hedgehog signaling pathway related to IKBIP expression in glioma.

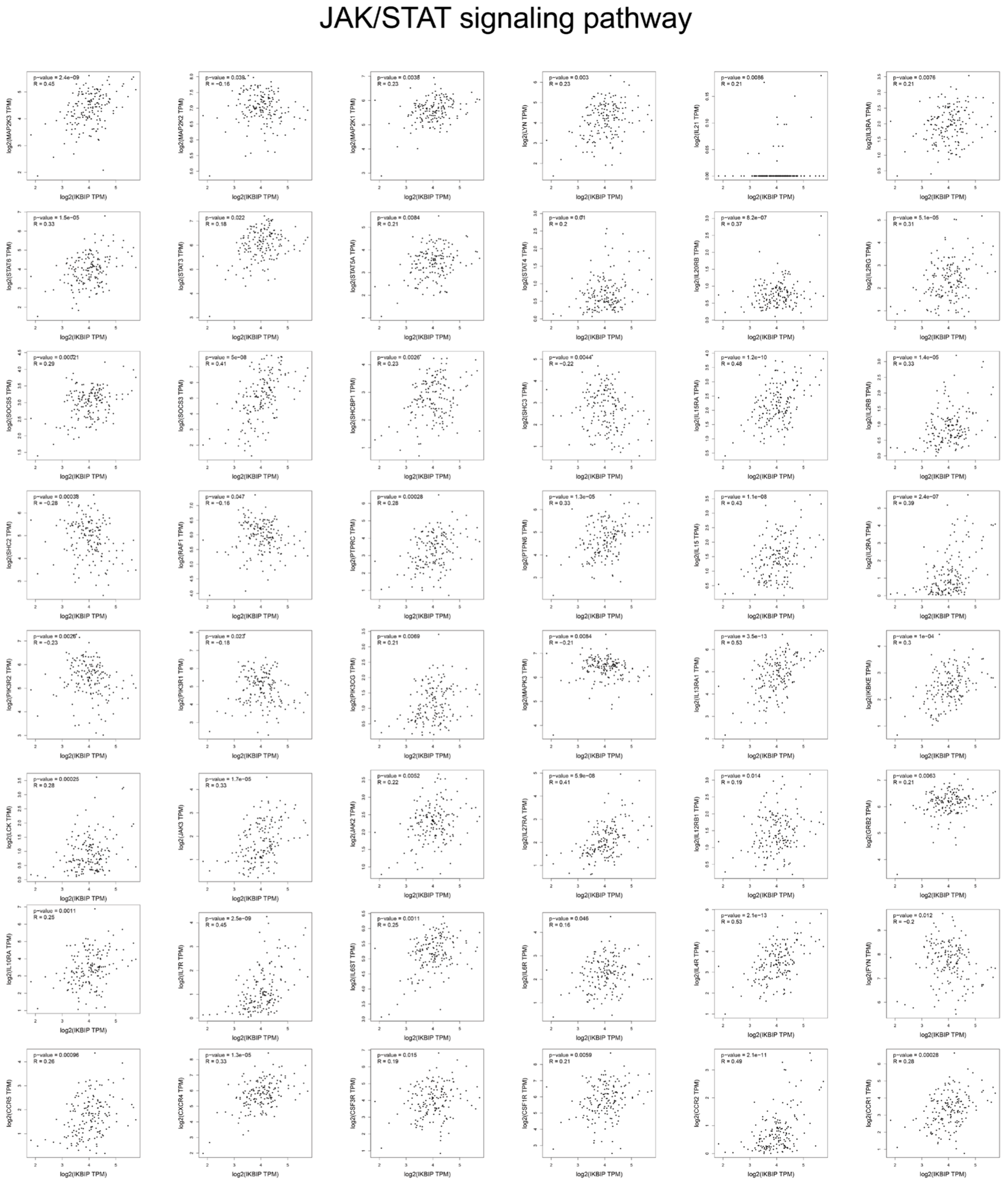

Supplementary Figure 8. Scatter plot shown genes in JAK/STAT signaling pathway related to IKBIP expression in glioma.

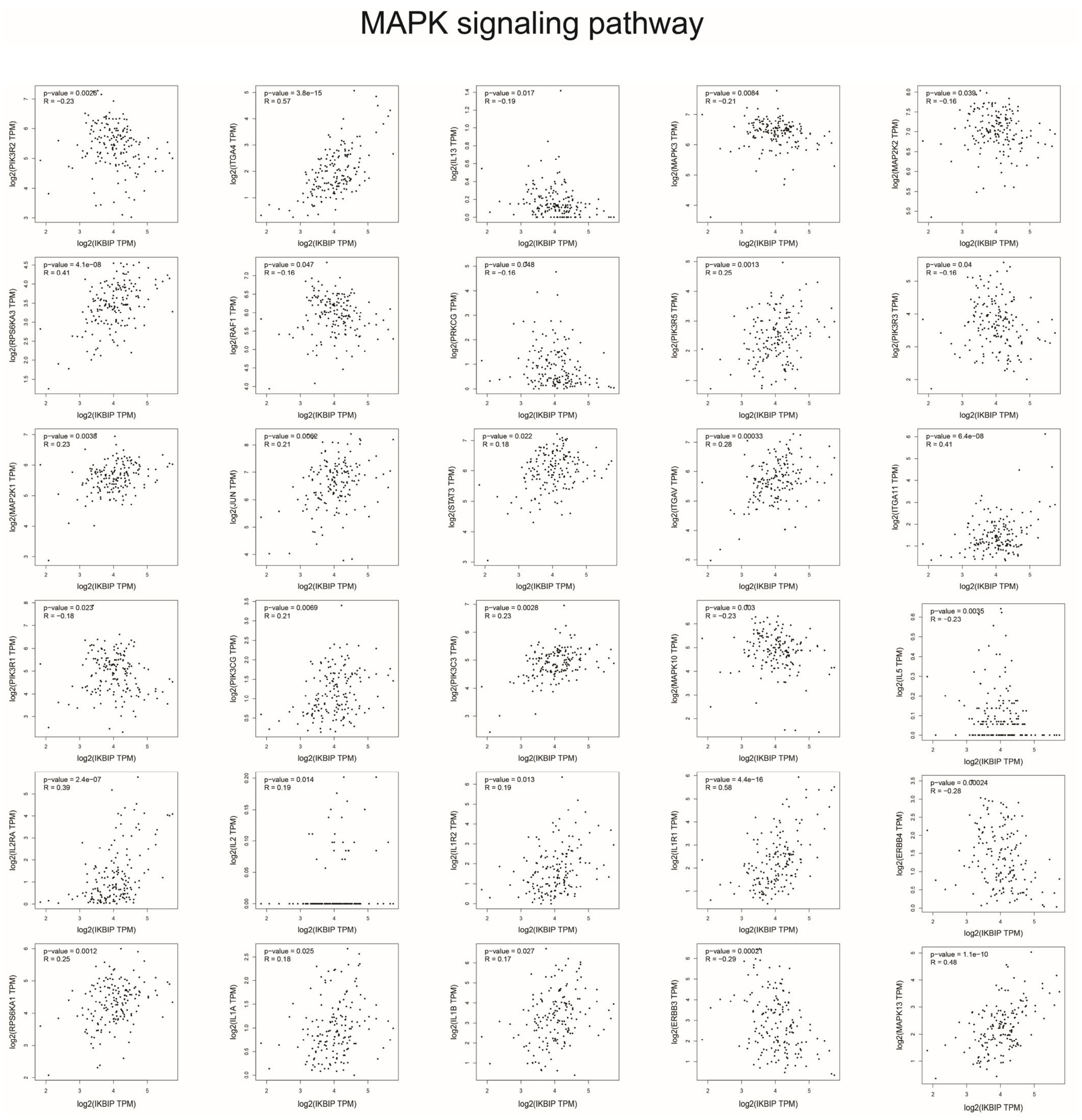

Supplementary Figure 9. Scatter plot shown genes in MAPK signaling pathway related to IKBIP expression in glioma.

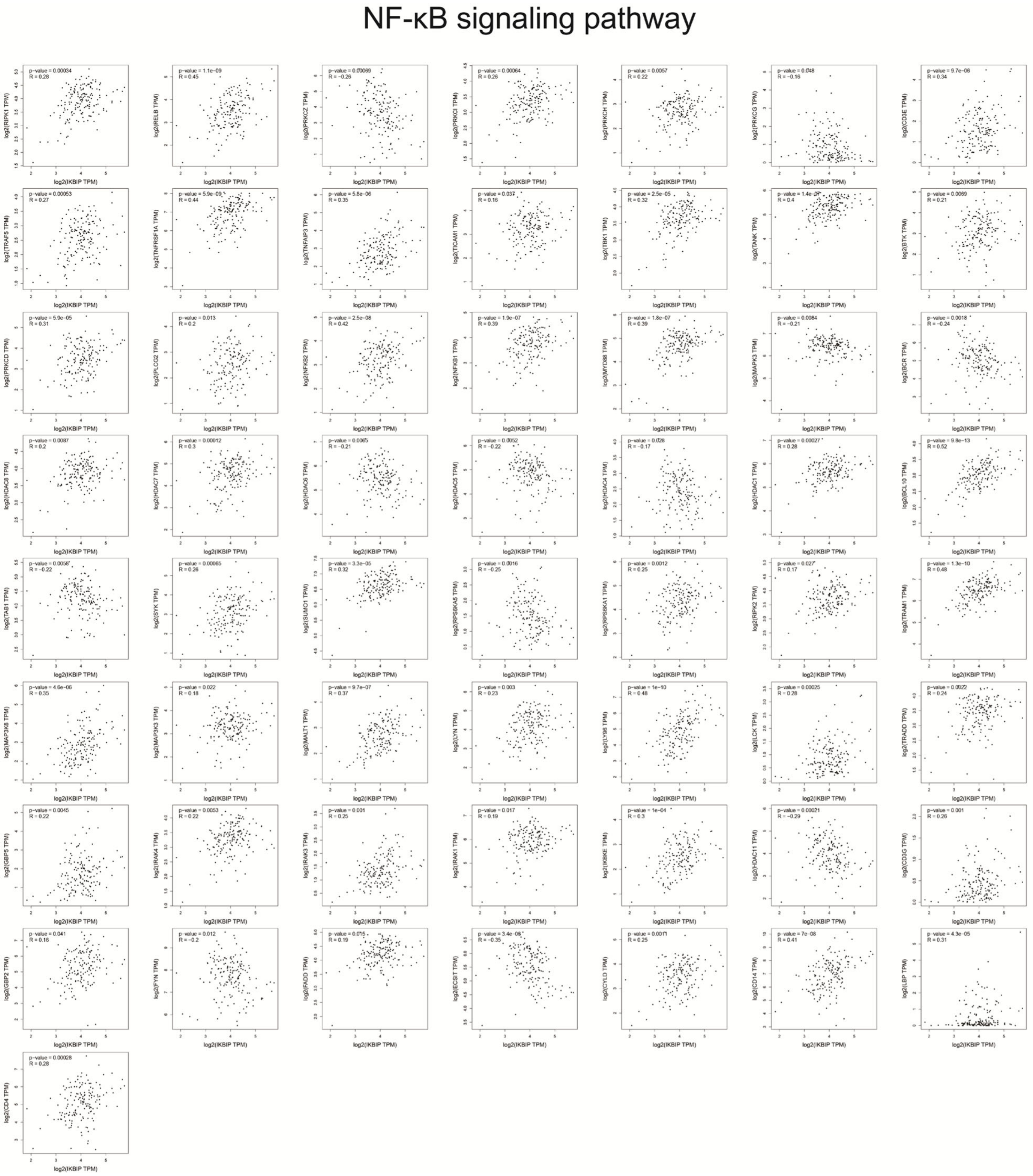

Supplementary Figure 10. Scatter plot shown genes in NF-κB signaling pathway related to IKBIP expression in glioma.

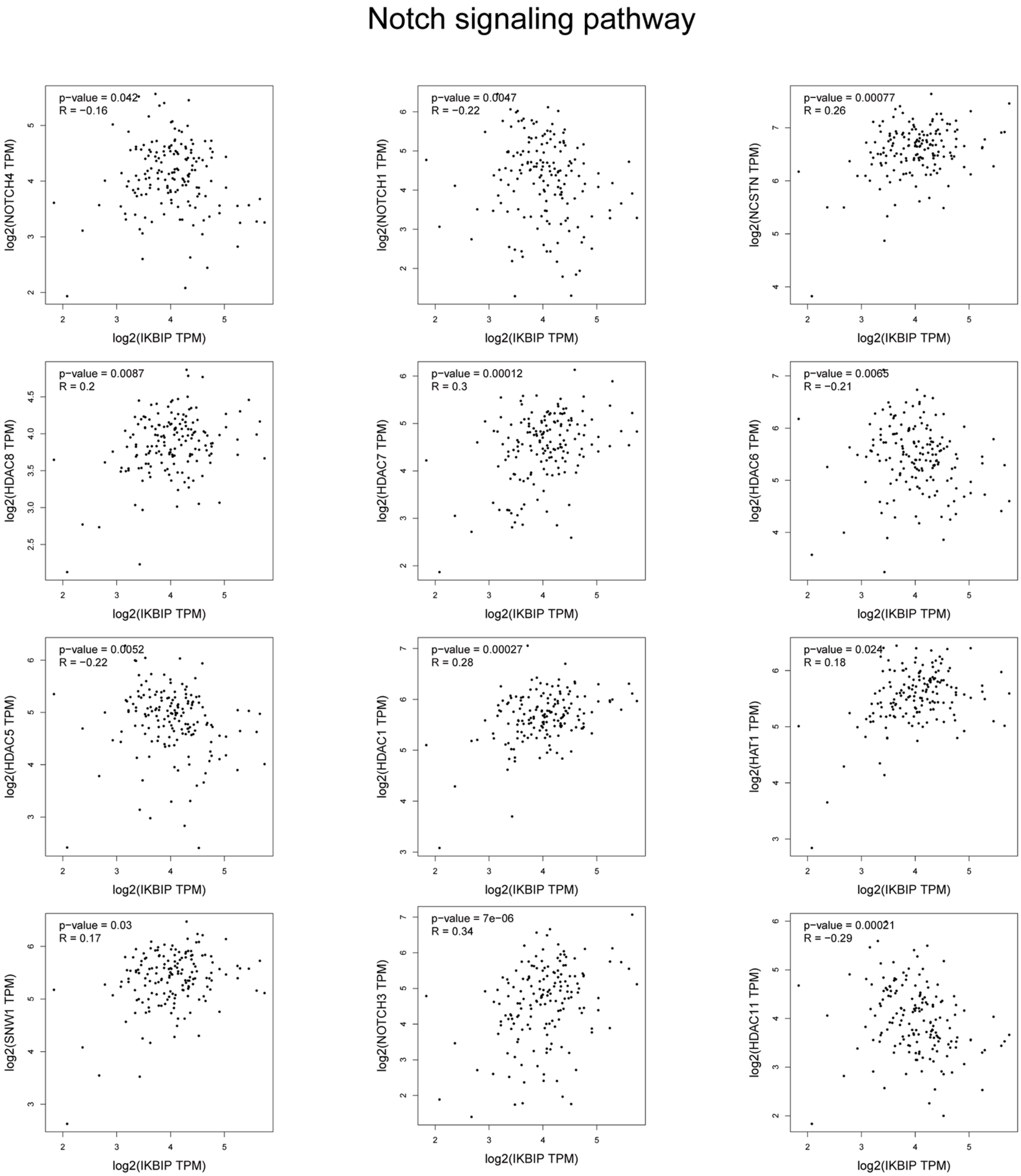

Supplementary Figure 11. Scatter plot shown genes in Notch signaling pathway related to IKBIP expression in glioma.

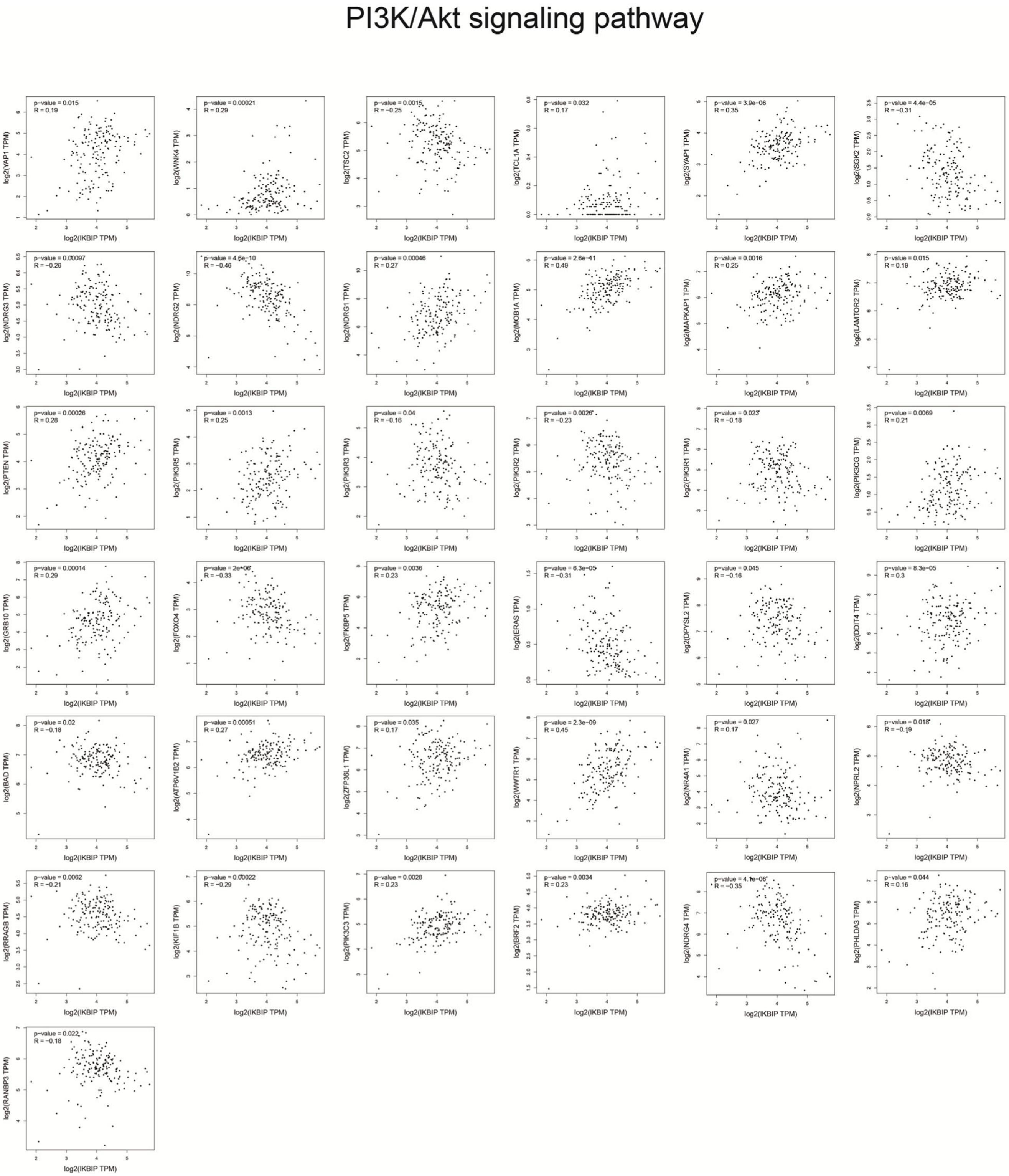

Supplementary Figure 12. Scatter plot shown genes in PI3K/Akt signaling pathway related to IKBIP expression in glioma.

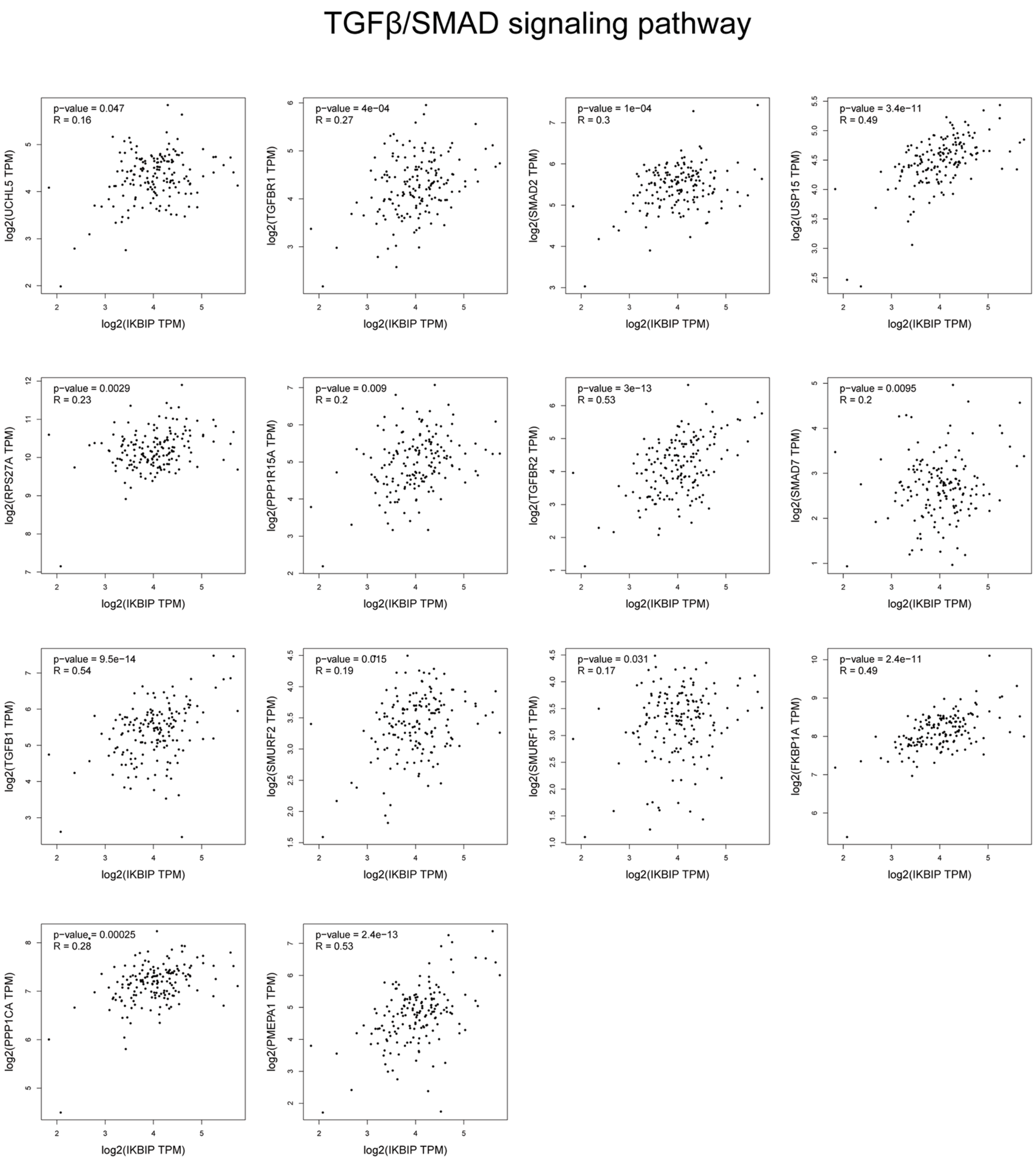

Supplementary Figure 13. Scatter plot shown genes in TGFβ/SMAD signaling pathway related to IKBIP expression in glioma.

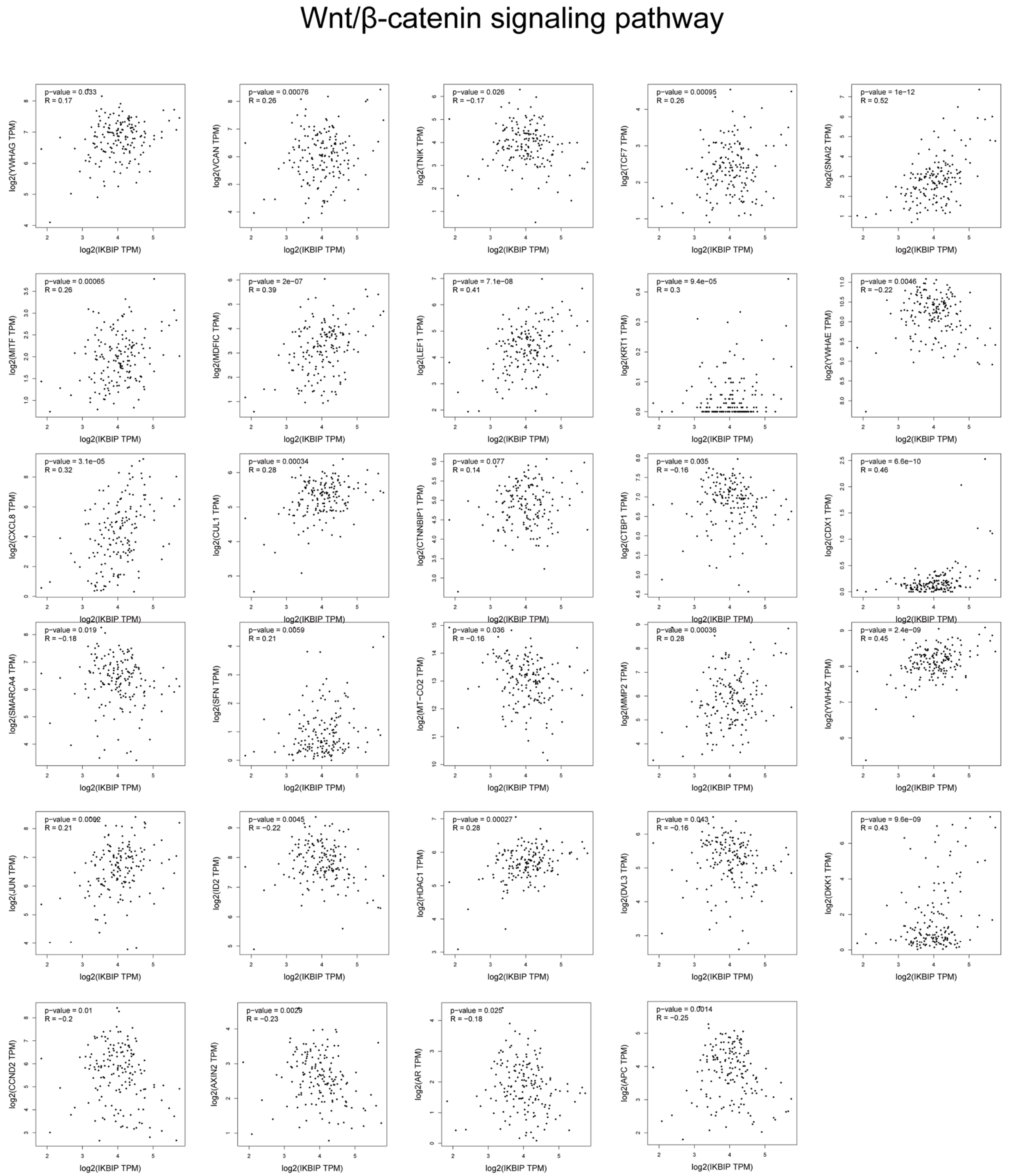

Supplementary Figure 14. Scatter plot shown genes in Wnt/β-catenin signaling pathway related to IKBIP expression in glioma.

signaling pathways

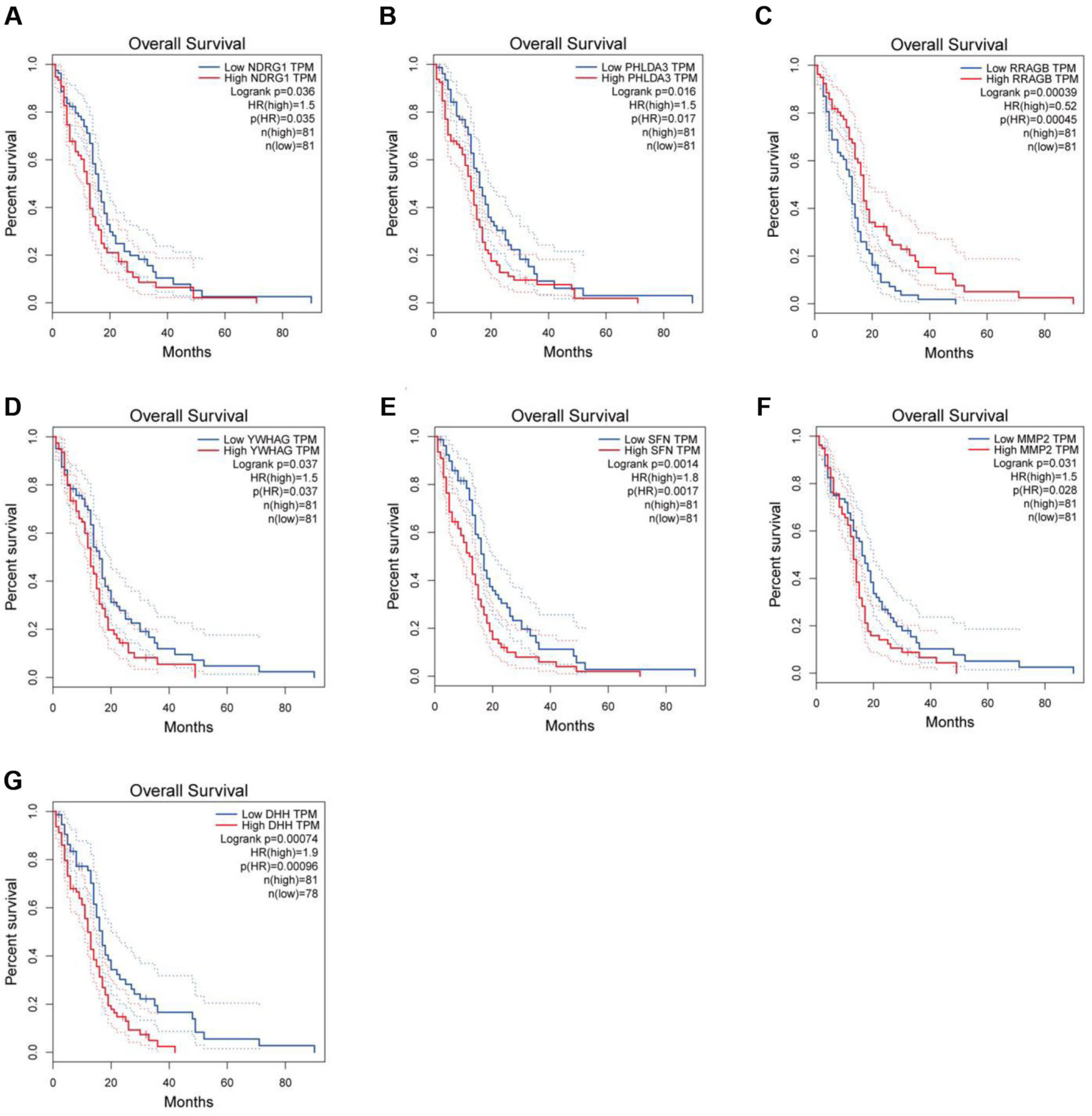

Supplementary Figure 15. Survival analysis shown the effect of genes related to IKBIP in the (**A**–**C**) PI3K/Akt signaling pathway, (**D**–**F**) Wnt/β-catenin signaling pathway, and (**G**) Hedgehog signaling pathway to glioma patients.
